# Self‐Assisted Charge Storage‐Release Mechanism Enabling Flexible Design of Biomimetic Triboelectric Nanogenerators

**DOI:** 10.1002/advs.202522836

**Published:** 2026-04-07

**Authors:** Hanpeng Gao, Yamin Hu, Li Li, Ana Sofia Oliveira Henriques Moita, Xi Wang, Zong Meng, Zhiwu Han, Yan Liu

**Affiliations:** ^1^ Hebei Key Laboratory of Measurement Technology and Instrumentation School of Electrical Engineering Yanshan University Qinhuangdao P. R. China; ^2^ IN+ ‐ Center for Innovation Technology and Policy Research Instituto Superior Técnico Universidade De Lisboa Lisboa Portugal; ^3^ School of Mechanical Engineering Yancheng Institute of Technology Yancheng P. R. China; ^4^ Key Laboratory of Bionic Engineering (Ministry of Education) Jilin University Changchun P. R. China; ^5^ Institute of Structured and Architected Materials Liaoning Academy of Materials Shenyan P. R. China

**Keywords:** breakdown threshold, lithium‐ion battery‐mimetic architecture, spontaneously regulating charge distribution

## Abstract

Triboelectric nanogenerators (TENGs) have attained a prominent position in the field of renewable energy conversion, and they exhibit immense promise for applications in self‐powered sensors and wearable devices. However, their practical power output is highly dependent on triboelectric effectiveness. While micro‐nano‐structured surfaces have been widely adopted in previous studies to enhance electrostatic effects, such architectures often lead to electric breakdown at the protruding sites, which adversely counteract the original intention. This work introduces a unique Lithium‐ion battery‐mimetic into a mimosa‐inspired TENG with a micro‐array surface. By spontaneously regulating charge distribution, it confines the interfacial electric field below the breakdown threshold, thus integrating high flexibility with outstanding electrical performance. Benefiting from a spontaneous charge self‐regulation mechanism, the TENG effectively suppresses air breakdown and achieves a high charge density of 396.50 µC m^−2^. Furthermore, this study demonstrates the practicality of the MLBA‐TENG in a long‐endurance intelligent insole for position monitoring, providing effective long‐term protection for special populations such as patients with Alzheimer's disease. The results validate the promising application prospects of the MLBA‐TENG in wearable intelligent devices, highlighting its potential to address critical needs in healthcare and mobile electronics.

## Graphical abstract

Inspired by the mimosa, this study develops a flexible triboelectric nanogenerator with a novel microneedle array and battery‐mimetic architecture. The device introduces a spontaneous charge self‐regulation mechanism that confines the electric field below the air breakdown threshold, and achieves an outstanding charge density of 396.50 µC m^−^^2^, integrating high flexibility with superior electrical output.

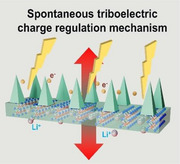

## 1 Introduction

The development of the Internet of Things (IoT) has drawn increasing attention to high sensing precision and self‐powering capabilities in sensors. Enabling sensors to efficiently harvest clean energy autonomously has become a critical challenge, where the development of novel materials emerges as a key solution [1, 2]. Human biomechanical energy, characterized by cleanliness, renewability, and continuous availability during movement, shows great potential for powering wearable sensors [3, 4, 5, 6]. For instance, Zhao et al. fabricated triboelectric nanogenerator (TENG)‐based textile pressure sensors featuring knitted, woven, and embroidered structures with machine washability and high air permeability for the first time via textile‐industry‐compatible technologies, uncovering the correlation between textile structure and sensor performance [7]. Among various technologies, TENGs have attracted widespread interest due to their simple structure and reliable performance [8, 9, 10]. Numerous studies have demonstrated their capability as energy harvesters for low‐power or intermittent electronic devices, revealing promising applications in self‐powered smart sensing systems [11, 12, 13]. Nevertheless, several limitations hinder the practical implementation of TENGs, particularly their low output power caused by insufficient surface charge density and rapid charge dissipation through air breakdown [14, 15, 16].

According to Maxwell's displacement current theory, the relationship between the ideal power density and surface charge density can be derived as: $P\propto \frac{\sigma^{2}fd}{\varepsilon }$. Where σ represents the surface charge density of the triboelectric layer, d denotes the separation distance between layers, and ε is the dielectric constant of air. Enhancing the surface charge density has become pivotal for improving triboelectric performance. However, the achievable charge density in triboelectric materials remains constrained by both the effective contact area and the air breakdown threshold [17, 18].

Based on the above analysis, a key research objective has been to maximize the triboelectric area within limited dimensions to boost charge density while simultaneously preventing charge dissipation caused by air breakdown [19, 20, 21, 22]. Current strategies to mitigate air breakdown in TENGs mainly involve four aspects: structurally minimizing the interlayer distance to reduce electric field strength while avoiding surface protrusions that could induce tip effects; selecting material pairs with closely matched triboelectric series positions to limit excessive charge accumulation; electrically designing active voltage‐limiting and charge‐shunting circuits; Increasing gas pressure to enhance electrical output performance [23, 24]; and environmentally enhancing insulation through inert gas filling or vacuum encapsulation [25, 26]. Unfortunately, according to the available report, these approaches frequently lead to substantial degradation of electrical output performance, increased fabrication complexity, and higher maintenance costs, thereby significantly restricting the practical application scenarios of TENG device [27, 28, 29].

Herein, we designed a smart insole capable of harvesting human mechanical energy, which integrates a bionic TENG, walk‐activated wake‐up system (WWS), and a GPS positioning system. This device aims to provide long‐term, effective, and stable protection for patients with Alzheimer's disease and related conditions. Inspired by the micro‐needle structure on the surface of mimosa leaves, the bionic TENG is endowed with a similar micro‐needle array architecture, significantly enhancing the effective contact during the contact–separation process of the nanogenerator. Furthermore, a lithium‐ion‐battery‐mimetic architecture is introduced to spontaneously regulate the charge density on the triboelectric surface, greatly mitigating the air breakdown phenomenon caused by abrupt contact and suppressing the escape of surface charges on the triboelectric layers. During TENG separation, the tribo‐positive layer actively releases electrons to balance surface potential, thereby transferring positive charges and maintaining interlayer electric field strength below the breakdown threshold. Upon re‐contact, the transferred positive charges are released back, achieving dual functions of charge accumulation and field strength suppression during separation. The charge transfer between triboelectric layers and graphite electrodes is mediated by lithium‐ion drift within gel electrolytes, which are recognized as a universal solution for addressing safety concerns and achieving high flexibility in wearable power systems [30, 31, 32]. The drift velocity of lithium ions in the electrolyte directly governs charge transfer rates across microneedle surfaces and determines the decay kinetics of interlayer electric fields [33, 34, 35].

Meanwhile, performance evaluations were conducted on microneedle arrays with lithium‐ion battery‐mimetic architecture TENG (MLBA‐TENG) and a normal TENG (N‐TENG). The results demonstrated that the MLBA‐TENG achieved superior electrical performance, with an open‐circuit voltage (V_oc_) of 190 V, a short‐circuit current (I_sc_) of 17.4 µA, and a short‐circuit charge quantity (Q_sc_) of 158.6 nC (396.50 µC m^−2^), corresponding to 1.72 times, 2.64 times, and 2.05 times those of the N‐TENG, respectively. These findings confirm that the unique lithium‐ion‐battery‐mimetic architecture of the MLBA‐TENG effectively suppresses the electric field intensity between the friction layers, thereby reducing performance degradation caused by charge leakage. The introduction of this specific vertical structure into TENGs across various application scenarios can significantly enhance their electrical performance. The strategy proposed in this study lays a foundation for the fabrication of high‐performance and high‐stability TENG devices and further expands the potential applications of TENG technology.

## 2 Results and Discussion

### 2.1 A Self‐Regulating Triboelectric Nanogenerator for Mitigated Air Breakdown

**FIGURE 1:**
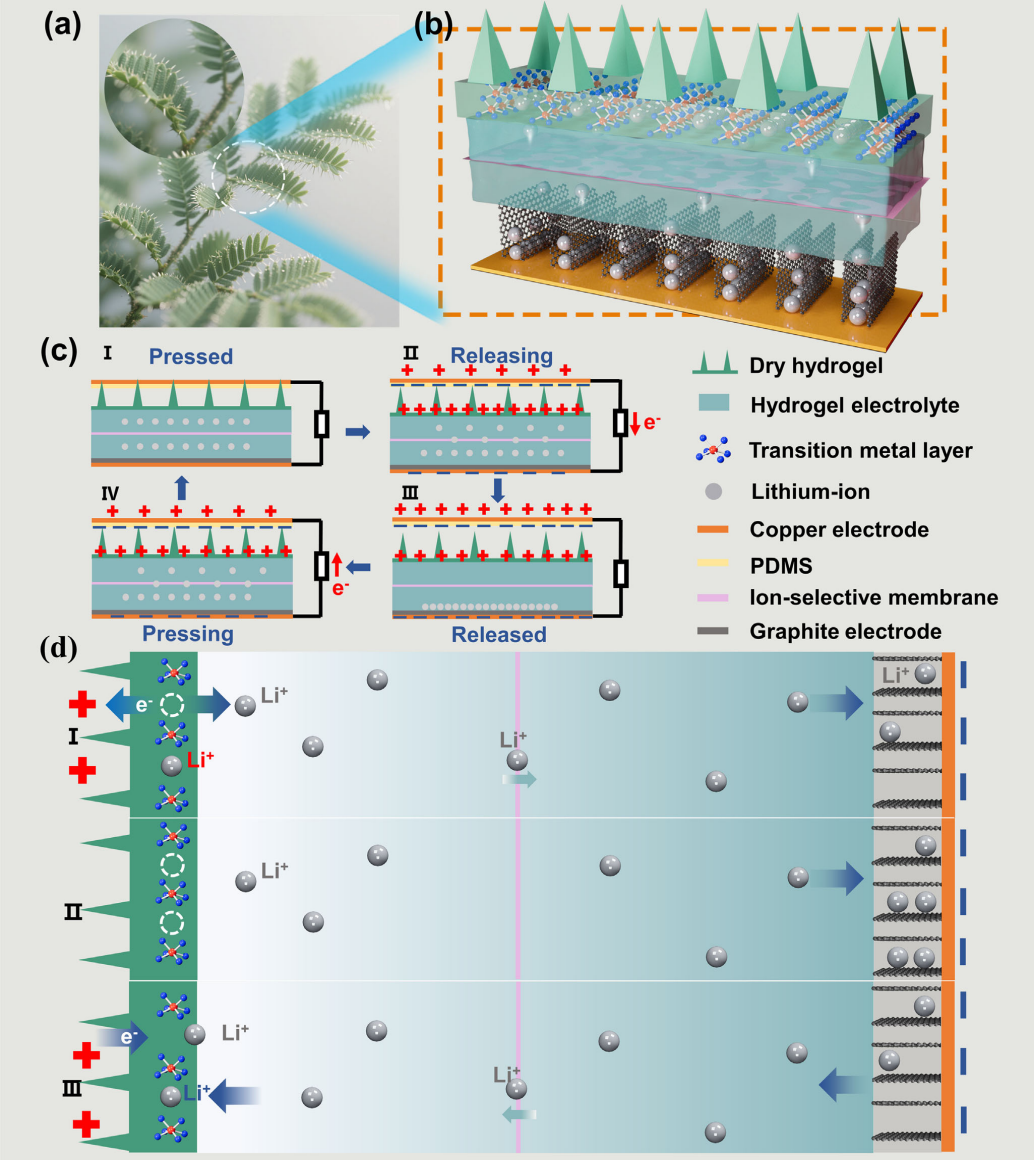
Structural design and working mechanism of the MLBA‐TENG. (a,b) Schematic illustration of the biomimetic MLBA‐TENG architecture: i) PVA film with surface microneedle structure incorporated with nickel cobalt manganese oxide (NCM)/carbon nanotube (CNT), ii) LiPF6/PVA electrolyte, iii) lithium‐ion separator, iv) graphite electrode, and v) copper foil current collector. (c) Operational mechanism during compression‐release cycles: I) Initial contact between PFOA‐modified PDMS and microneedle layer; II) Electron transfer from PVA to PDMS upon compression; III) Induced charge generation during separation; IV) Electron flow through external circuit. (d) Self‐regulating charge balance mechanism: I) Electron loss occurs at the NCM phase; II) lithium‐ion migration through electrolyte; III) lithium‐ion returns from graphite to NCM.

Inspired by the mimosa pudica microstructure, a triboelectric nanogenerator (TENG) featuring surface microneedle arrays with a lithium‐ion battery‐mimetic architecture was designed. Microneedle‐arrays with lithium‐ion battery‐mimetic architecture TENG (MLBA‐TENG) features a unique vertically multilayered lithium battery‐like structure, with the tribo‐positive electrode comprising a microneedle layer, a lithium‐ion electrolyte layer, a lithium‐ion separator layer, a graphite electrode layer, and a copper foil electrode layer (Figure 1a,b). During compression, the perfluorooctanoic acid (PFOA)‐treated PDMS exhibits strong electron‐capturing capability, facilitating charge transfer upon contact between the tribo‐positive and tribo‐negative electrodes [36, 37].

The PVA loses electrons and becomes positively charged, while the PDMS gains electrons and becomes negatively charged. During the release process, induced charges are generated in the copper foil, and electrons flow from the copper foil atop the PDMS to the bottom PVA electrode through the external circuit (Figure 1c). The calculation was performed based on the microneedle dimensions (length: 800 µm, base area: 360 × 360 µm) and array configuration (25 × 25), microneedle layers enlarge triboelectric contact area by 2.44 times relative to conventional planar structures, markedly boosting the hydrogel film triboelectric performance at identical dimensions. Nickel cobalt manganese oxide (NCM) and carbon nanotube (CNT) powders embedded in the microneedle layer mediate bidirectional transfer for tribo‐generated charges across the film. A LiPF_6_/PVA composite electrolyte containing 10 wt.% LiPF_6_ enables reversible Li^+^ ions shuttling across the separator membrane [38, 39].

When positive charges are generated on the upper surface of the microneedle layer, the induced negative charges on the copper foil flow toward the graphite electrode through the external circuit. Simultaneously, electrons are released from the transition metal elements in the microneedle layer and migrate to the upper surface to neutralize the positive charges, thereby reducing the electric field strength between the tribo‐positive and tribo‐negative layers. This self‐regulating mechanism confines the electric field strength below the breakdown threshold, minimizing charge leakage caused by air breakdown. Notably, this modulation of interlayer electric field strength occurs spontaneously and is cyclically sustainable. Meanwhile, lithium ions detach from the transition metal layer and enter the electrolyte [40, 41, 42]. Upon electron loss from the NCM oxide framework, Li^+^ ions are extracted from the transition metal layer and diffuse into the electrolyte. The electrolyte's sufficient Li^+^ ions inventory enables reversible intercalation/deintercalation at the graphite electrode during charge/discharge cycling (Figure 1d) [43, 44, 45, 46]. This process enables the storage and release of surface charges on the triboelectric layer, while restricting the surface electric field strength below the breakdown threshold [47]. The PVA/NCM hybrid system exhibits notable advantages, as PVA can effectively disperse and anchor NCM particles, enhancing the structural stability of the electrode. It also facilitates efficient Li^+^ transport across the matrix while accommodating the volume variation of NCM during charge‐discharge cycles, contributing to improved cycling stability and electrochemical performance of the battery.

### 2.2 Optimized NCM Oxide for High‐Performance TENG via Charge Transport

**FIGURE 2:**
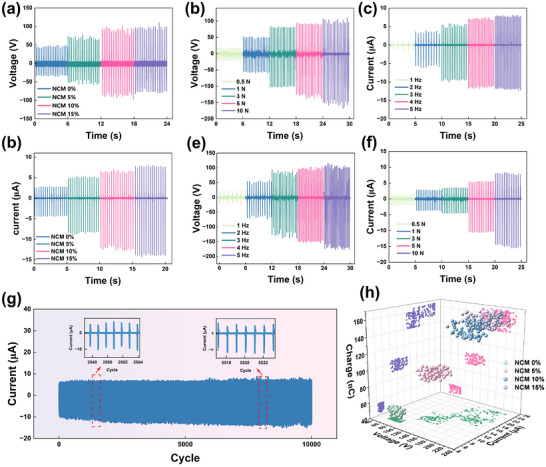
Electrical performance characterization and optimization of MLBA‐TENG. (a) Output voltage comparison of MLBA‐TENGs with different NCM doping concentrations (0–15 wt.%) in PVA microneedle films under 5 N force at 3 Hz. (b) Pressure‐dependent open‐circuit voltage (V_oc_) as force increases from 0.5 to 10 N (3 Hz). (c) Frequency‐dependent (V_oc_) improvement from 1 to 5 Hz at 5 N. (d–f), Corresponding short‐circuits current (I_sc_) measurements: NCM concentration effect (7.1–22 µA), pressure response, frequency response. (g) Durability test over 10 000 cycles showing stable signal retention. (h) Comprehensive performance matrix comparing voltage, current, and charge transfer across all tested parameters.

The MLBA‐TENG was constructed using PVA/NCM composite films with varying NCM concentrations (0, 5, 10, and 15 wt.%) as the tribo‐positive layer. Electrical characterization was performed on MLBA‐TENG devices with an active area of 4 cm^2^ (2 × 2 cm) under contact‐separation mode using a linear reciprocating motor, applying a 5 N force at 3 Hz frequency to measure output voltage, short‐circuit current, and transferred charge. The pristine PVA microneedle hydrogel tribo‐positive electrode (0 wt.% NCM) exhibited an open‐circuit voltage (V_oc_) of 70 V and a short‐circuit current (I_sc_) of 7.1 µA. In contrast, MLBA‐TENGs incorporating NCM at different concentrations demonstrated significantly enhanced electrical performance. The optimal device, fabricated with 10 wt.% NCM‐doped PVA paired with a PDMS tribo‐negative layer, achieved a V_oc_ of 185 V and an I_sc_ of 17.4 µA (Figure 2a,d). The performance enhancement mechanism can be attributed to electron donation from NCM during triboelectrification. Upon friction, NCM supplies electrons to neutralize positive charges on the tribo‐positive surface, thereby lowering its surface potential. This effect confines the interfacial electric field strength below the air breakdown threshold, effectively suppressing charge dissipation caused by air ionization. Concurrently, lithium ions deintercalated from the transition metal oxide layers of NCM and migrate into the electrolyte. These ions subsequently diffuse across the separator and intercalate into the graphite electrode layer. This dual mechanism facilitates efficient charge transfer from the tribo‐positive surface to the graphite electrode, significantly mitigating charge loss and improving overall electrical output. Notably, when the NCM concentration reached 15 wt.%, no further performance improvement was observed. Moreover, excessive NCM loading reduced the flexibility of the PVA microneedle film and considerably increased fabrication complexity (Figure S5). A systematic comparison of the voltage, current, and charge transfer characteristics for MLBA‐TENGs with different NCM loadings (0–15 wt.%) under 5 N force at 3 Hz operation revealed substantial performance enhancement across all metrics in the 0–10 wt.% range, while negligible differences were observed between 10 and 15 wt.% devices (Figure 2h).

Further, the influence of two key external factors, applied pressure and operational frequency, on the electrical output of the MLBA‐TENG were evaluated. The device was fabricated with copper foil electrodes and designed with an active area of 4 cm^2^ (2 × 2 cm). Under a fixed frequency of 3 Hz, the open‐circuit voltage (V_oc_) demonstrated a significant increase from 37.6 V to 256 V as the applied pressure was gradually raised from 0.5 to 10 N (Figure 2b). Correspondingly, the short‐circuit current (I_sc_) exhibited similar enhancement, increasing from 1.96 to 19.49 µA (Figure 2e). When maintaining a constant pressure of 5 N while varying the frequency from 1 to 5 Hz, the V_oc_ showed progressive improvement from 43.6 to 284 V, with the I_sc_ simultaneously increasing from 3.02 to 23.3 µA (Figure 2c–f). Notably, the rate of enhancement in both V_oc_ and I_sc_ became less pronounced when the frequency exceeded 3 Hz, suggesting a saturation effect at higher operational frequencies. To assess practical applicability, a rigorous durability test involving 10 000 continuous contact‐separation cycles at 5 N and 3 Hz was conducted (Figure 2g). The triboelectric output signals remained remarkably stable throughout this extended testing period, confirming the exceptional mechanical durability and operational stability of our MLBA‐TENG design.

The electrical output performance was systematically evaluated under specific mechanical conditions (3 Hz frequency) using a MLBA‐TENG with 2 × 2 cm area. By employing external load resistors ranging from 10 Ω to 1000 MΩ, and the output voltage and current characteristics were measured, from which the power output was calculated (Figure S6a). The results revealed a clear load‐dependent behavior: the output voltage exhibited a progressive increase with rising load resistance, while the current demonstrated an inverse relationship, gradually decreasing with higher resistances. The instantaneous peak power density, calculated using the equation P = UI/S (where U represents voltage, I denotes current, and S is the active area), reached its maximum value of 2.36 W m^−2^ when the external load resistance was 5 MΩ (Figure S6b). Overall, these findings demonstrate that the proposed strategy is effective in enhancing TENG performance, offering a novel design perspective for future TENG development.

### 2.3 Self‐Regulating Triboelectric Nanogenerator Mimics Lithium Battery Dynamics for Sustained High‐Output Energy Harvesting

**FIGURE 3:**
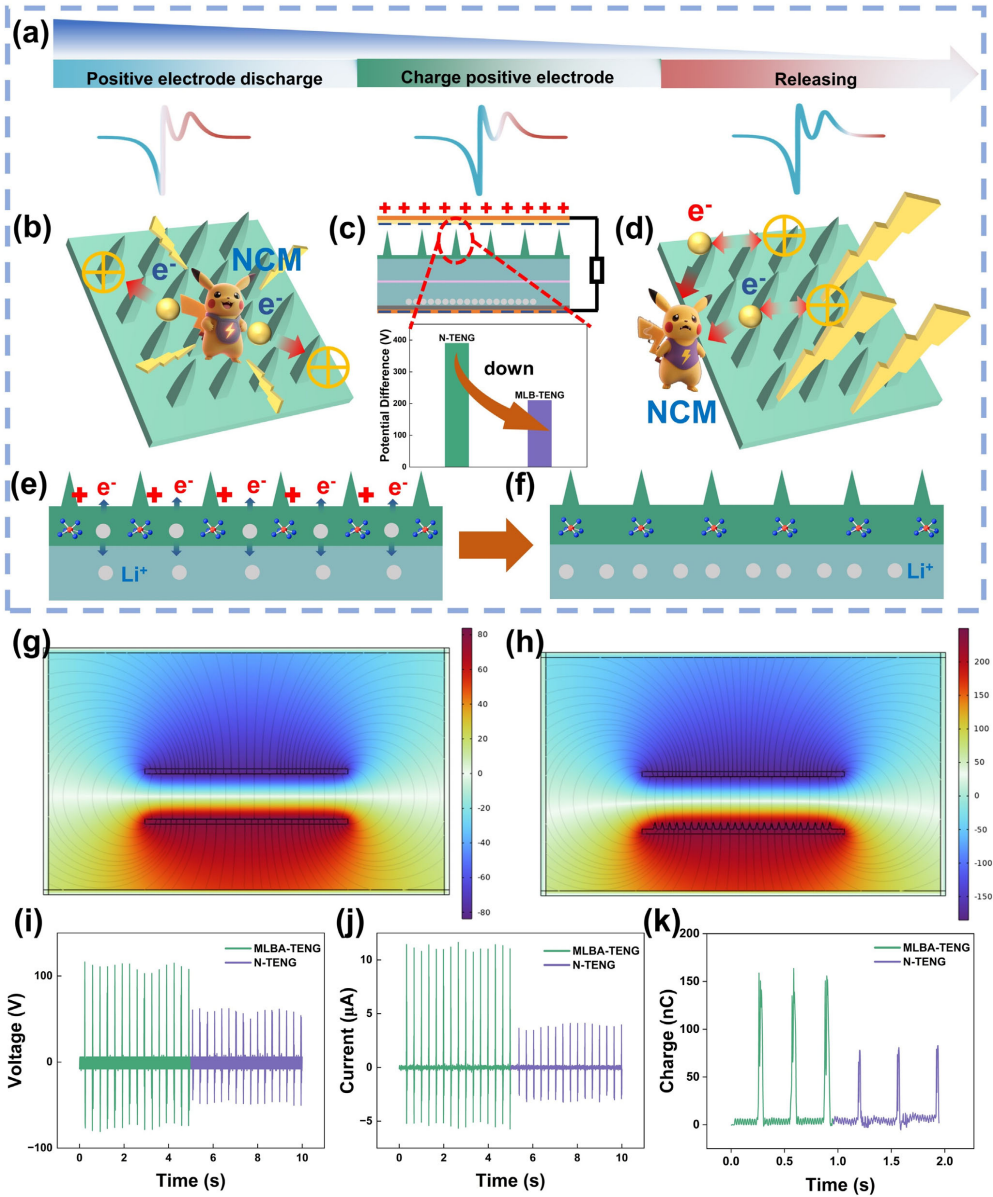
Charge regulation mechanism and performance advantages of MLBA‐TENG. (a) Schematic of the three‐peak signal generation mechanism during cyclic pressing. (b) NCM oxidation supplies electrons to balance tribo‐induced positive charges on microneedle surfaces. (c) Surface potential measurements showing 68% reduction in potential difference for MLBA‐TENG vs. N‐TENG (n = 5, ^***^ *p*<0.001). (d) Charge redistribution process during compression: positive charges (red) re‐emerge on microneedle surfaces. (e,f) Schematic of lithium‐ion deintercalation from transition metal layers. (g,h) COMSOL simulations comparing triboelectric potential. (i–k) Under identical conditions (5 N, 3 Hz), the MLBA‐TENG demonstrates significantly enhanced performance compared to the N‐TENG.

Further analysis of the structural advantages of the MLBA‐TENG, the electric field distribution around the triboelectric layers was simulated (Figure S7), and the elemental distribution of NCM was characterized using X‐ray energy‐dispersive spectroscopy (EDS) (Figures S9–S11). These investigations reveal further insights into the internal kinetic mechanisms of the lithium‐ion‐battery‐mimetic architecture and its effect on the electrical output of TENGs. Due to the lithium‐battery‐mimetic vertical structure of the triboelectric positive electrode, a unique three‐peak triboelectric signal can be observed under continuous cyclic pressing (Figure S12). This phenomenon occurs because the lithium‐like battery structure completes charge storage during the previous cycle and continues to reversely release charge in the pressure release phase of the subsequent cycle, thereby affecting the peak value generated by the separation of the triboelectric. Simultaneously, the electric field produced by the separation of the triboelectric layers enables the lithium‐battery‐mimetic structure to recharge in this phase, repeating its effect in the subsequent cycle (Figure 3a). Lithium‐ion battery‐mimetic architecture autonomously regulates surface charge density of PVA microneedle film by releasing and absorbing charges, reducing interlayer electric field during the separation‐release process of the MLBA‐TENG, and thereby capturing escaped charges caused by air breakdown.

When the microneedle film generates positive charges through friction, the lithium atoms doped in the film lose electrons. Due to the high conductivity of the metal and carbon nanotube powder incorporated into the microneedle film, electrons migrate to the film's surface to neutralize the triboelectric positive charges, thereby weakening the interfacial electric field and significantly reducing charge escape caused by air breakdown. Measurement of the interlayer electric field strength demonstrated that the MLBA‐TENG shows a markedly reduced surface potential difference compared to the N‐TENG (Figure 3b,c). When the friction layers are compressed again, the positive charges are re‐released onto the surface of the microneedle film (Figure 3d).

The neutralized positive charges do not disappear but instead drift in the electrolyte as lithium ions and embed into the graphite interlayer (the negative charges cannot penetrate due to the ion‐selective membrane) [48, 49]. When the charging voltage dissipates, the lithium ions de‐intercalate, and the microneedle film releases positive charges again, while electrons in the graphite layer return to the upper copper foil through the external circuit (Figure S13). The deintercalation process of lithium ions from the transition metal layer is illustrated in Figure 3e,f.

Based on the parameters provided in Table S1, a triboelectric model was developed using COMSOL Multiphysics software, incorporating the charge density, area, pressing/release distance, and material properties of the two triboelectric layers. The microneedle structure significantly increases the effective contact area during friction, which promotes electron capture and storage, thereby increasing the surface charge density. The triboelectric potentials measured for the PDMS film paired with the flat and microneedle‐structured PVA films were 155 and 372 V, respectively. These results demonstrate that both surface functionalization and structural design play critical roles in modulating the triboelectric properties of the MLBA‐TENG, leading to a substantial increase in the interlayer triboelectric potential [50] (Figure 3g,h). The dynamic process can be seen in Movie S1.

To highlight the structural superiority of the MLBA‐TENG, we compared its electrical performance with that of the N‐TENG using devices with an identical size of 2 × 2 cm^2^. A linear reciprocating motor and pressure gauge were employed to ensure constant frequency and pressure during all measurements. Both nanogenerators were tested under the same conditions: 5 N impact force at 3 Hz. The MLBA‐TENG delivered output voltages of 190 and 110 V, short‐circuit currents of 17.4 and 6.6 µA, and transferred charges of 158.6 nC (396.50 µC m^−^^2^) and 77.5 nC (193.75 µC m^−^^2^), respectively, which are markedly higher than those of the N‐TENG (Figure 3i–k). Such remarkable enhancements in electrical output can be mainly attributed to the unique vertical structure inspired by lithium‐ion batteries. This distinctive architecture effectively confines the interlayer electric field and strongly suppresses charge escape, thereby endowing the MLBA‐TENG with significant performance advantages over the conventional N‐TENG. Figure S14 presents a comparison of charge density between the MLBA‐TENG and other TENGs reported in previous studies. It can be clearly observed that the charge density of MLBA‐TENG has been improved from 32 to 332 µC m^−2^ compared with other TENGs.

Based on the excellent performance of MLBA‐TENG, the operational mechanism of the lithium‐ion‐battery‐mimetic structure was further investigated from the perspective of its autonomous charge regulation. Tribo‐positive electrode consists of a PVA‐based composite layer embedded with NCM/CNT, LiPF_6_/PVA electrolyte, separator, graphite electrode, and copper current collector, enabling bidirectional charge transfer and reversible Li^+^ shuttling. The SOC of the graphite electrode, which serves as the lithium ions reservoir, is governed by the lithium ions intercalation/deintercalation equilibrium:

(1) $$SOC_{graphite}\;\left(t\right)=\frac{c_{s}^{graphite}\left(t\right)}{c_{s,max}^{graphite}}$$

where $c_{s}^{graphite}$ is the time‐dependent lithium ion concentration in the graphite layer, and $c_{s,max}^{graphite}$ is its theoretical maximum capacity. During compression, the triboelectric charge generation induces electron flow from the NCM layer to neutralize the PVA‐derived positive surface charges, concurrently triggering lithium ions extraction from the NCM lattice. The lithium‐ion concentration distribution within the NCM particles is governed by the following partial differential equation (PDE):

(2) $$\frac{\partial c_{s}^{NCM}\left(r,t\right)}{\partial t}=D_{s}^{NCM}\nabla^{2}c_{s}^{NCM}-\;\frac{j_{TENG}\left(t\right)}{nF}$$

Here, $D_{s}^{NCM}$ (∼10^−14^ m^2^ s^−1^) is the solid‐state diffusion coefficient of lithium ions in NCM, and *j_TENG_* is the tribocurrent density, $c_{s}^{NCM}$ is the time (t) and radial position (r) dependent lithium‐ion concentration within NCM particles (unit: mol m^−3^), n denotes the number of electrons transferred per reactive event (typically n = 1 for NCM delithiation: LiNi_0.6_Co_0.2_Mn_0.2_O_2_ → Li_1‐X_Ni_0.6_Co_0.2_Mn_0.2_O_2_ + xLi^+^ + xe^−^), F denotes the faraday constant, linking charge to molar quantity of Li^+^. The local SOC of the NCM surface (θ_*NCM*_​) reflects the degree of charge compensation:

(3) $$\theta_{NCM}\left(r,t\right)=\frac{c_{s}^{NCM}\left(r,t\right)}{c_{s,max}^{NCM}}$$

Here, $c_{s,max}^{NCM}$ is the maximum Li^+^ concentration in NCM (∼50 000 mol m^−3^ for NCM622). The tribo‐induced electric field (*E_filed_* ​) is dynamically stabilized below the air breakdown threshold by the NCM‐to‐graphite Li^+^ migration. When θ_*NCM*_ decreases due to electron donation, Li^+^ detaches from the NCM lattice and diffuses toward the graphite electrode, raising *SOC_graphite_* ​. This feedback loop ensures:

(4) $$E_{field}=\frac{\sigma_{+}\left(t\right)}{\varepsilon_{0}\varepsilon_{r}}<\;E_{breakdown}\;\left(\sigma_{+}\propto 1-\theta_{NCM}\right)$$

### 2.4 Electrochemical Simulation of Lithium‐ion Battery‐mimetic Architecture and Equivalent Circuit Modeling

**FIGURE 4:**
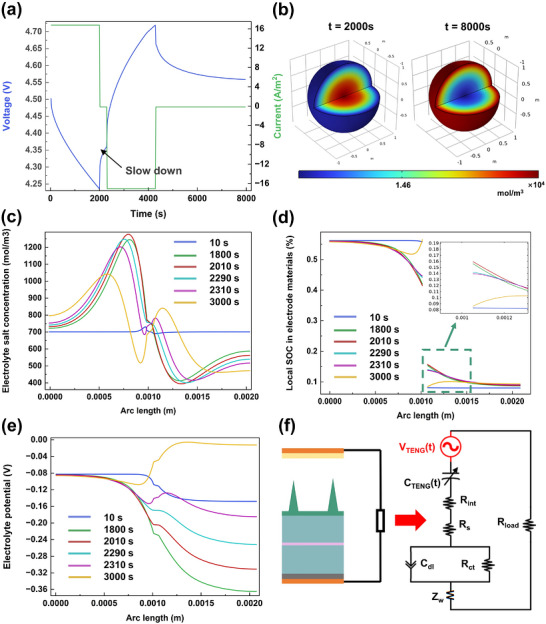
Electrochemical performance analysis of lithium‐battery‐mimetic structure via 1D COMSOL simulation. (a) Voltage and current profiles during discharge–charge–discharge cycling. (b) Simulated Li^+^ concentration gradients in solid‐phase electrodes. (c) Electrolyte salt concentration profiles cross positions in the 1D lithium battery‐analogous model at t = 10, 1800, 2010, 2290, 2310, and 3000 s. (d) Local state‐of‐charge (SOC) distribution in electrode materials at different times. (e) Time‐resolved potential profiles at multiple positions within the lithium‐battery‐mimetic structure. (f) Equivalent circuit diagram of the MLBA‐TENG under external load conditions.

To further investigate the electrochemical kinetics of the lithium‐ion battery‐analogous structure under an applied electric field, a 1D battery model with a lithium‐battery‐mimetic structure was established using COMSOL Multiphysics software based on the parameters provided in Table S2. A simulation test of 2000 s discharge–300 s rest–2000 s charge was conducted, and the voltage‐current‐time variation curves of the battery were plotted (Figure 4a). The charge–discharge curves demonstrated that this structure exhibits electrical properties similar to those of common lithium batteries. Figure 4b presents a 3D model of the solid‐phase lithium‐ion concentration gradient. After discharging (at 8000 s), a gradual decrease in concentration from the cathode to the anode is observed, whereas the opposite trend is exhibited after charging (at 2000 s).

By extracting the electrolyte salt concentration at various positions of the model at 10, 1800, 2010, 2290, 2310, and 3000 s, as well as the local state of charge and internal potential of the electrodes, the movement patterns of lithium ions during the charge–discharge process were analyzed. This analysis verified the successful charge embedding and de‐embedding within the anode and cathode of the lithium‐battery‐mimetic architecture. It further elucidated the drift dynamics of lithium ions in the electrolyte and the effect of reaction kinetics on the electrochemical potential following the application and removal of an external electric field (Figure 4c–e).

Figure 4f shows the equivalent circuit model of the MLBA‐TENG when connected to an external resistive load. In this model, the friction‐negative electrode and the intermediate gap are represented as an alternating voltage source (V_TENG_) in series with a variable capacitor (C_TENG_) and an internal resistance (R_int_). The friction‐positive electrode, corresponding to the lithium‐battery‐inspired structure, is modeled as the combined impedance of the separator and electrolyte (R_s_), connected in series with a parallel branch consisting of charge transfer resistance (R_ct_) and electric double‐layer capacitance (C_dl_), followed by a Warburg impedance (Z_w_) in series. Figure S15 shows the equivalent circuit diagram of the N‐TENG. The establishment of an equivalent circuit model facilitates elucidation of TENG operation mechanisms, optimization of output characteristics, guidance for impedance matching, enhancement of energy harvesting efficiency, and advancement of practical applications. In the lithium battery‐ mimetic architecture, the drift velocity of lithium ions shows little dependence on the amplitude of voltage variations at the triboelectric points from 10 to 100 V. Even without applying a high external voltage or extended periods, the drift velocity can rapidly reach saturation, as illustrated in Figure S8.

### 2.5 A Mechanically Triggered Wake‐up System with Adaptive Power Management

**FIGURE 5:**
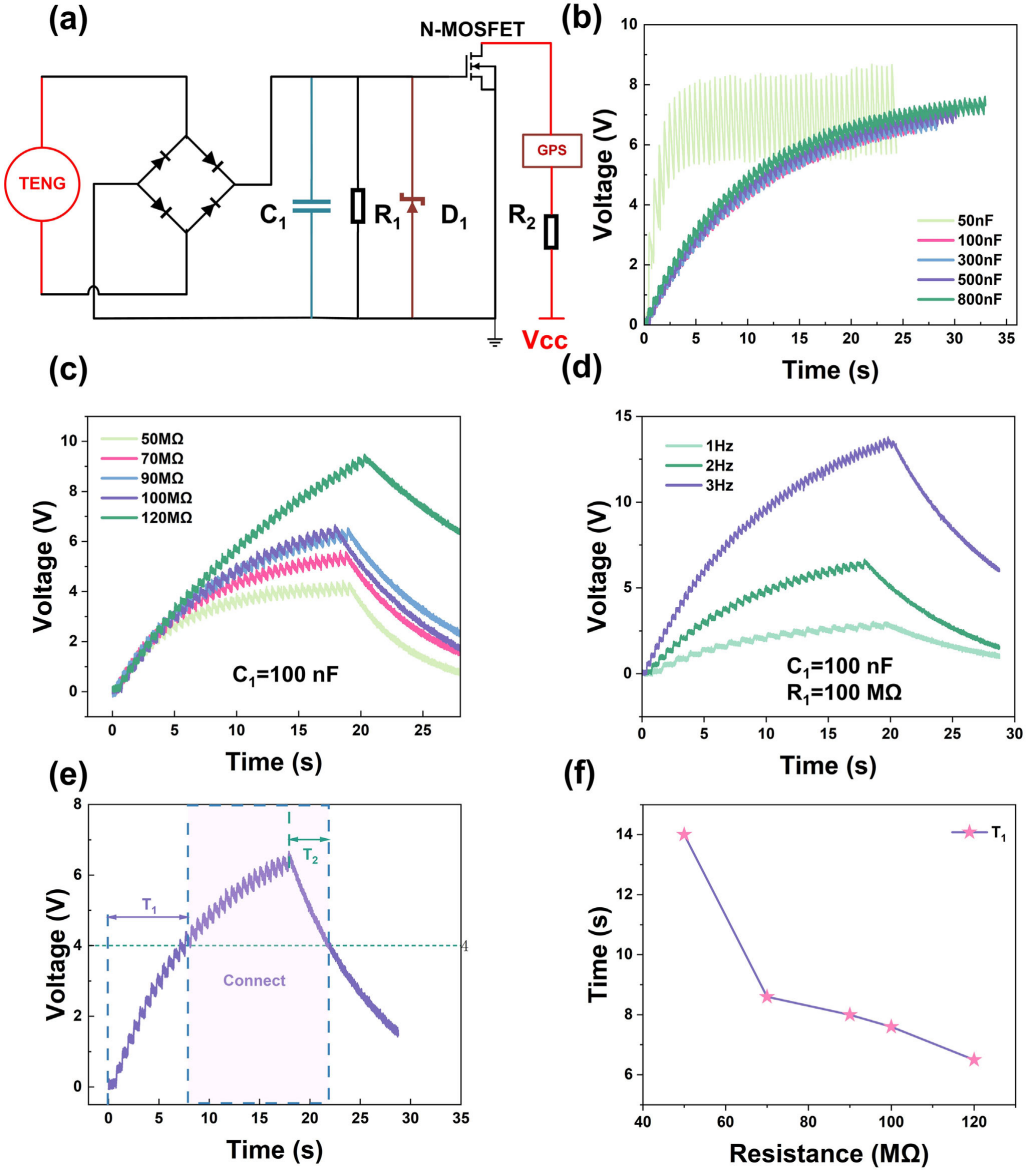
Circuit design and performance evaluation of the Walk‐Activated Wake‐up System (WWS). (a) Circuit diagram of WWS showing: (i) MLBA‐TENG energy harvesting, (ii) bridge rectifier, (iii) storage capacitor (C_1_), and (iv) voltage‐triggered switch (Zener D_1_ + N‐MOSFET). (b) Capacitor charging tests (100—800 nF) demonstrating consistent charging rates. (c) Saturation voltage vs discharge resistance (50–120 MΩ). (d) Time‐domain charging curve (C_1_ = 100 nF, R_1_ = 100 MΩ) under 3 N, 2 Hz excitation. (e,f) Wake‐up characteristics: Activation time (T_1_=7.6 s to reach 4 V) and sleep delay (T_2_=3.8 s from 6.67 to 4 V). T_1_ decreases exponentially with increasing resistance (50–120 MΩ).

Here, an integrated triboelectric circuit system named the Walk‐Activated Wake‐up System (WWS) is presented. The output of MLBA‐TENG is rectified via a bridge rectifier and then fed into the WWS, which consists of a storage capacitor (C_1_), a Zener diode (D_1_), an N‐MOSFET, and resistors R_1_, R_2_. When the wearer moves, electricity harvested by the MLBA‐TENG is contained in capacitor C_1_. Resistor R_1_ provides a discharge path for C_1_ to prevent accidental circuit activation when the wearer stops moving. The Zener diode D_1_ offers overvoltage protection for the N‐MOSFET, while resistor R_2_ acts as a current‐limiting resistor (Figure 5a).

Under a 5 N force applied at 3 Hz, an MLBA‐TENG with an effective contact area of 2 × 2 cm^2^ was employed to charge capacitors of various capacitances. The results demonstrate that the charging rates for capacitors ranging from 100 to 800 nF show no significant difference, indicating the MLBA‐TENG's capability to charge large‐capacity capacitors (Figure 5b). When external circuits with different resistances (50–120 MΩ) were connected as discharge paths for C_1_, the saturation voltage of C_1_ increased with higher resistance values of R_1_ (Figure 5c).

Under mechanical excitation at 1–3 Hz frequency and 3 N pressure, the MLBA‐TENG charged the external circuit (C_1_ = 100 nF, R_1_ = 100 MΩ) as shown in the corresponding charging curve Figure 5d. Under continuous mechanical input at 2 Hz frequency and 3 N pressure, the MLBA‐TENG charged the external circuit (C_1_ = 100 nF, R_1_ = 100 MΩ) to the turn‐on voltage (4 V) in 7.6 s (wake‐up time, T_1_). After energy input ceased, the stored voltage decreased from 6.67 to 4 V in 3.8 s (decay time, T_2_), at which point the GPS module's power was cut off, entering sleep mode. The system would be reactivated upon the wearer's next movement (Figure 5e). When C_1_ was fixed at 100 nF, T_1_ was investigated under different discharge resistances (50–120 MΩ). The results show that T_1_ gradually decreased as the resistance increased (Figure 5f). The foot‐strike energy harvesting experiment for the MLBA‐TENG is demonstrated in Movie S2.

### 2.6 Self‐powered Wearable Sensing System with Motion‐activated Localization

**FIGURE 6:**
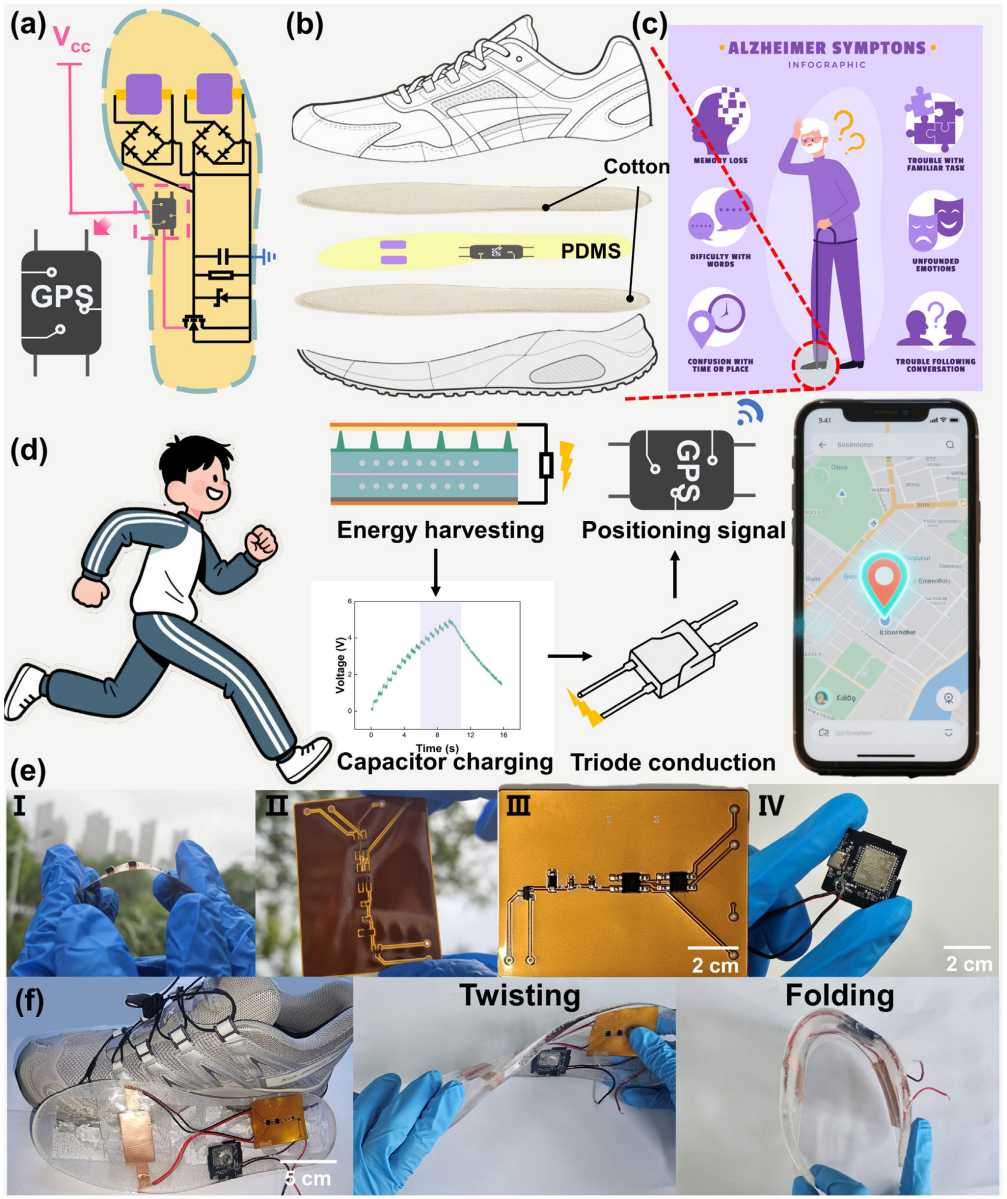
Wearable smart insole application powered by MLBA‐TENG. (a) System integration: Two parallel‐connected MLBA‐TENG units (4 cm^2^ each) with wake‐up circuit and GPS chip, encapsulated in PDMS. (b) Smart insole integrated between conventional shoe insoles. (c) Shoes integrated with the smart insole enable long‐term, stable monitoring for special populations such as Alzheimer's patients. (d) working mechanism: Mechanical energy → MLBA‐TENG harvesting → capacitor charging → GPS activation (threshold: 4 V). (e) Flexible printed circuit (FPCB). (f) Mechanical robustness tests: Twisting (180°) and folding without performance degradation.

Based on the outstanding energy harvesting performance of the MLBA‐TENG and the specific needs of particular populations, such as individuals with Alzheimer's disease, WWS was developed. This system demonstrates the application potential of MLBA‐TENG as a wearable smart sensor. As illustrated in Figure 6a, two MLBA‐TENG units (2 cm × 2 cm) were rectified and connected in parallel, then integrated with the wake‐up circuit and a GPS chip. The system was encapsulated in PDMS to form an intelligent insole capable of detecting human motion and activating a positioning system. Figure 6b shows that the smart insole can be packaged like a conventional insole and placed in any shoe of the corresponding size.

A key advantage of this system is that the circuit remains inactive for extended periods when the wearer is stationary, significantly reducing power consumption. However, once the wearer moves, the system harvests energy from body motion to activate the GPS function. Compared to traditional positioning devices with the same power supply capacity, this system achieves significantly prolonged operational endurance (Figure 6c). This feature is particularly beneficial for long‐term monitoring of special populations, such as Alzheimer's patients, who require continuous location tracking. Since frequent battery replacement is unnecessary, the system provides more stable and durable protection for users.

The operational principle of the system is illustrated in Figure 6d: movement of the wearer triggers the conversion of mechanical energy generated by foot pressure into electrical energy via the MLBA‐TENG. After rectification, the energy charges the capacitor. As movement continues, it accumulates sufficient charge, and once the voltage reaches the N‐MOSFET's threshold, the GPS module is activated, transmitting real‐time location data to a smartphone (Figure S16). The positioning system of the smart insole is illustrated in Movie S3. GPS module parameters are listed in Table S3.

The wake‐up circuit system, fabricated on a flexible printed circuit board (FPCB) via immersion gold plating, is shown in Figure 6e (dimensions: 1700 mil × 2145 mil). FPCBs provide notable benefits for wearable devices owing to their light weight, conformability, and flexibility, key requirements for wearable devices. The flexible substrate (polyimide) allows repeated bending, folding, and even twisting, with a minimum bending radius below 0.1 mm. With a typical thickness of 50–100 µm and a weight only 1/3–1/5 that of traditional PCBs, the FPCB can be designed in curved or irregular shapes based on ergonomic needs. Finally, Figure 6f shows the smart insole, which exhibits flexibility comparable to that of ordinary household insoles, enabling twisting and folding without compromising functionality or durability–thanks to the integration of the flexible circuit.

## 3 Conclusion

In conclusion, we present a mimosa‐inspired microneedle‐arrays with lithium‐ion battery‐mimetic architecture triboelectric nanogenerators (MLBA‐TENG) that addresses two critical limitations of conventional triboelectric nanogenerators (TENGs): low surface charge density and charge dissipation via air breakdown, through a combination of bioinspired design and rational material engineering. This device achieves high flexibility and enhanced output performance by leveraging a unique longitudinal architecture that spontaneously modulates charge distribution during the friction‐separation cycle, confining interlayer electric field intensity below the breakdown threshold. The MLBA‐TENG achieves the most balanced overall performance at an NCM content of 10%, delivering the maximum open‐circuit voltage, short‐circuit current, and output power density reached 284 V, 23.3 µA, and 2.36 W m^−2^, respectively, its electrical performance is 1.72, 2.64, and 2.05 times that of a conventional triboelectric nanogenerator without such a composite structure (N‐TENG), respectively. This study demonstrates the practical viability of the MLBA‐TENG through its implementation in a smart insole system designed for continuous position monitoring, particularly targeting vulnerable groups, including Alzheimer's patients. The successful deployment of this self‐powered monitoring solution confirms the device's readiness for real‐world wearable applications. Through synergistic enhancement of charge density (achieved via the 2.44 times increased contact area from microneedle architecture) and effective suppression of air breakdown (enabled by spontaneous charge redistribution), compared with current research, our work represents a significant advancement in TENG technology. These innovations collectively overcome fundamental limitations that have hindered practical adoption, paving the way for broader implementation of high‐performance triboelectric systems in next‐generation wearable electronics and self‐powered sensor networks.

## 4 Experimental Section

### 4.1 Fabrication of the Triboelectric Negative Electrode

The polydimethylsiloxane (PDMS) based MXene composite film was prepared using a doctor‐blading technique. First, the PDMS (Sylgard 184, Dow Corning) was thoroughly mixed with the curing agent at a 10:1 weight ratio. Subsequently, 2 wt.% of MXene (Ti_3_C_2_T_x_) flakes were gradually incorporated into the PDMS mixture and dispersed uniformly via 30 min mechanical stirring followed by 15 min ultrasonication (100 W, 40 kHz) to achieve a homogeneous suspension. Degassing of the mixture was conducted under vacuum for 20 min while maintaining room temperature. The viscous solution was then uniformly coated onto a cleaned glass substrate using a blade with a gap height of 0.5 mm (Figure S1). Subsequently, the film was cured at 80°C for 2 h in a convection oven to facilitate complete cross‐linking. The freestanding MXene/PDMS nanocomposite film was finally detached from the glass substrate, producing a flexible and homogeneous film with well‐controlled thickness. The as‐prepared MXene/PDMS composite film was subjected to surface chemical modification to enhance its triboelectric properties. The film was immersed in a 5 wt.% perfluorooctanoic acid (PFOA, 90%, MERYER) ethanol solution for 5 h at room temperature to facilitate the formation of a low‐surface‐energy fluorinated layer. After treatment, the film was carefully extracted using tweezers, and remained PFOA and solvent were removed by thorough rinsing with deionized water. Subsequently, the sample was dried at 60°C for 1 h in a convection oven to eliminate residual solvent and ensure stable functionalization.

### 4.2 Fabrication of the Triboelectric Positive Electrode

A 20 wt.% polyvinyl alcohol (PVA, P815608, MACKLIN) hydrogel solution was synthesized by dispersing 20 g of PVA powder in 80 g of deionized water, followed by constant magnetic stirring at 200 rpm and heating in a water bath for 2 h (Figure S2). The resulting solution was then degassed under vacuum for 5 min to remove air bubbles. Nickel cobalt manganese oxide (NCM)/ carbon nanotube (CNT)/PVA composite mixtures with varying NCM concentrations (5, 10, and 15 wt.%) were prepared using a 20 wt.% PVA solution as the matrix, while maintaining a constant CNT concentration of 1 wt.%. The homogeneous mixtures were carefully injected into 20 × 20 mm^2^ microneedle molds and subsequently cured at 30°C for 8 h (Figure S3). This fabrication protocol enabled the systematic investigation of NCM content effects on the composite's properties while ensuring consistent CNT dispersion throughout the PVA hydrogel network. As shown in Figure S4, the fabricated microneedle array exhibits a well‐defined structure.

### 4.3 Electrical Performance Characterization

The experiments were performed under atmospheric pressure (1 atm = 101.325 kPa). The electrical output characteristics of the triboelectric nanogenerators (TENGs), including both microneedle‐arrays with lithium‐ion battery‐mimetic architecture TENG (MLBA‐TENG) and normal TENG (N‐TENG) configurations, were comprehensively evaluated using specialized instrumentation: the output current and charge were quantified using a Keithley 6517B electrometer, the corresponding voltage was monitored with an oscilloscope. All measurement data were acquired through an NI‐6218 data acquisition system. Current signals and charge transfer quantities were measured using an NI‐6218 data acquisition system.

## Conflicts of Interest

The authors declare no conflict of interest.

## Supporting information




**Supporting File 1**: advs75086‐sup‐0001‐SuppMat.docx.

**Supporting File 2**: advs75086‐sup‐0002‐MovieS1.mp4.

**Supporting File 3**: advs75086‐sup‐0003‐MovieS2.mp4.

**Supporting File 4**: advs75086‐sup‐0004‐MovieS3.mp4.

## Data Availability

The data that support the findings of this study are available from the corresponding author upon reasonable request.
