# Supplementary material for: Self‐Assisted Charge Storage‐Release Mechanism Enabling Flexible Design of Biomimetic Triboelectric Nanogenerators
Source: Adv Sci (Weinh). 2026 Apr 7;13(34):e22836. doi: 10.1002/advs.202522836 (PMC13285128; doi:10.1002/advs.202522836)
Supplement: Supplementary file 1 — Supporting File 1: advs75086‐sup‐0001‐SuppMat.docx. [file ADVS-13-e22836-s002.docx]

**Self-assisted charge storage-release mechanism enabling flexible design of biomimetic triboelectric nanogenerators**

Hanpeng Gao^a^, Yamin Hu^a^, Li Li^a^, Ana Sofia Oliveira Henriques Moita^b^, Xi Wang^c^, Zong Meng^a^, Zhiwu Han^d^, Yan Liu*^d, e^

*^a^ Hebei Key Laboratory of Measurement Technology and Instrumentation, School of Electrical Engineering, Yanshan University, Qinhuangdao, 066004, P. R. China;*

*^b^ IN+ - Center for Innovation, Technology and Policy Research, Instituto Superior Técnico, Universidade de Lisboa, Av. Rovisco Pais, Lisboa, 1049-001, Portugal;*

*^c^ School of Mechanical Engineering, Yancheng Institute of Technology, Yancheng 224051, P. R. China*

*^d^ Key Laboratory of Bionic Engineering (Ministry of Education), Jilin University, Changchun 130022, P. R. China;*

*^e^ Institute of Structured and Architected Materials, Liaoning Academy of Materials, Shenyang 110167, China*

Corresponding author: Yan Liu,

Email address: lyyw@jlu.edu.cn

**Contents**

1. Test method and characterization 3

2. Electrical Performance Analysis of MLBA-TENG 6

3. Structural Analysis of MLBA-TENG 8

4. Electrochemical Simulation and Analysis 14

5. Performance Simulation 16

# Test method and characterization

- 1. ***Characterization of Positive and Negative Triboelectric Layers.***

Polydimethylsiloxane (PDMS) has been widely adopted as the negative triboelectric material in triboelectric nanogenerators (TENGs) due to its exceptional dielectric properties and mechanical flexibility. This material exhibits a strong electron affinity, which facilitates efficient electron capture and stabilization, thereby effectively boosting the charge output density of TENG devices ^[1]^. Furthermore, its low surface energy minimizes interfacial adhesion, ensuring efficient separation of triboelectric layers. However, the rapid surface charge decay of PDMS substantially limits its long-term stability.

Surface fluorination effectively addresses this limitation through two primary mechanisms: (1) The incorporation of fluorine atoms (Pauling Scale electronegativity: 3.98) creates deeper surface potential wells, extending charge retention time by more than threefold (from 12 h to 40 h); (2) The fluorinated layer reduces the contact angle below 110°, thereby optimizing triboelectric contact efficiency. This modification preserves the intrinsic advantages of PDMS while establishing a stable fluorinated siloxane (Si-O-F) interfacial layer, which effectively suppresses charge leakage induced by environmental humidity. Therefore, surface fluorination serves as a key strategy to effectively improve the environmental adaptability and service stability of PDMS‑based TENGs ^[2]^.


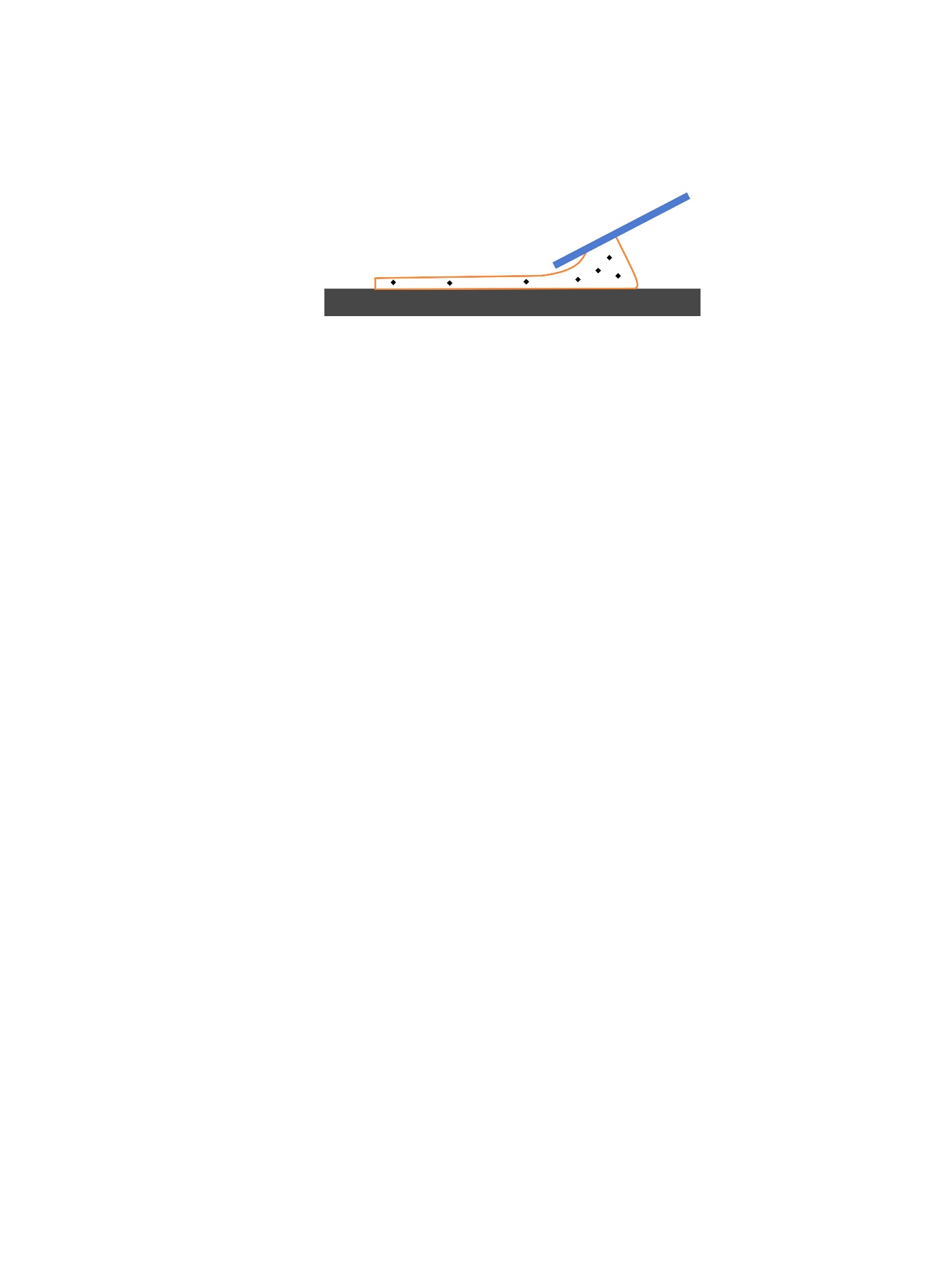


**Fig. S1**. Fabrication of MXene/PDMS composite films via doctor-blading method.

Polyvinyl alcohol (PVA), as a positively charged polymer, exhibits excellent electron-donating capability in the triboelectric series. When paired with negative triboelectric materials such as PDMS, it significantly enhances the output performance of TENGs. Compared to conventional positive electrode materials, PVA offers two key advantages: (1) Its abundant hydroxyl (-OH) groups enable efficient charge transfer and favor the generation of high-density surface charges during contact electrification ^[3]^; (2) its tunable porous structure not only increases the effective contact area for triboelectrification but also provides favorable channels for lithium-ion transport.

Moreover, the selection of the tribo-positive material is not limited to a specific system; any polymeric matrix that fulfills the following criteria can be adopted: (1) possessing interconnected porosity to facilitate ion migration, (2) exhibiting a suitable position in the triboelectric series to ensure efficient charge generation, and (3) functioning as an effective binder to integrate conductive or active fillers.

Materials such as polyethylene oxide (PEO) and polyacrylamide (PAM) also basically meet the requirements of the triboelectric positive electrode. However, compared with alternative polymer binders such as polyethylene oxide (PEO) and polyacrylamide (PAM), poly(vinyl alcohol) (PVA) exhibits distinct advantages in the construction of high-performance hydrogel electrodes for bio-inspired lithium-ion batteries, even though PEO, PAM, and other hydrogel systems can theoretically provide porous frameworks and adjustable triboelectric characteristics that meet the basic requirements of binder functionality and triboelectric polarity. First, PVA possesses mature and well-established processing routes, enabling stable and reproducible fabrication of porous, flexible hydrogel matrices that not only serve as effective binders for conductive fillers (CNTs) and active NCM particles but also accommodate the structural and ionic dynamics required by the bio-inspired lithium-ion battery architecture, whereas PEO often suffers from relatively low mechanical strength and high crystallinity at room temperature that restrict its structural stability during repeated cycling, and PAM, while highly hydrophilic, is prone to excessive swelling and structural collapse in liquid electrolytes, leading to unstable electrode integrity and deteriorated long-term cycling performance. Second, PVA exhibits controllable and stable swelling behavior in electrolyte environments, a crucial feature for maintaining electrode porosity and efficient Li⁺ transport without severe dimensional changes, which ensures that the hydrogel matrix can accommodate volume variations of active NCM materials during charge–discharge processes while permitting the shuttling of Li⁺ ions across the electrolyte-separator interface. Third, PVA demonstrates excellent compatibility with embedded electroactive fillers including CNTs and NCM particles, acting as a robust yet flexible binder that effectively holds conductive and active materials together while maintaining the permeability of the polymer network to Li⁺ ions, in contrast to PEO which typically requires high-temperature operation or complex modification to achieve satisfactory ionic conductivity and PAM which may introduce additional interface impedance due to its strong water retention and unstable gel structure in organic electrolytes. Collectively, PVA provides the optimal balance of processability, structural stability, controllable swelling, and interfacial compatibility among the candidate polymers, making it the most suitable binder matrix for the proposed bio-inspired lithium-ion battery architecture.

This study proposes a multifunctional composite material fabricated by incorporating lithium nickel cobalt manganese oxide (NCM) and carbon nanotubes (CNTs) into a PVA matrix. In TENG applications, the introduction of NCM and CNT particles, combined with LiPF_6_ electrolyte and graphite electrodes, not only enhances electron transfer efficiency but also suppresses air breakdown during charge accumulation, offering a novel approach for improving TENG performance^[4]^. The fabrication process of the composite material is illustrated in **Fig. S2**, while the preparation of the microneedle membrane is detailed in Fig. The microneedle structure of the composite material is shown in **Fig. S4**.


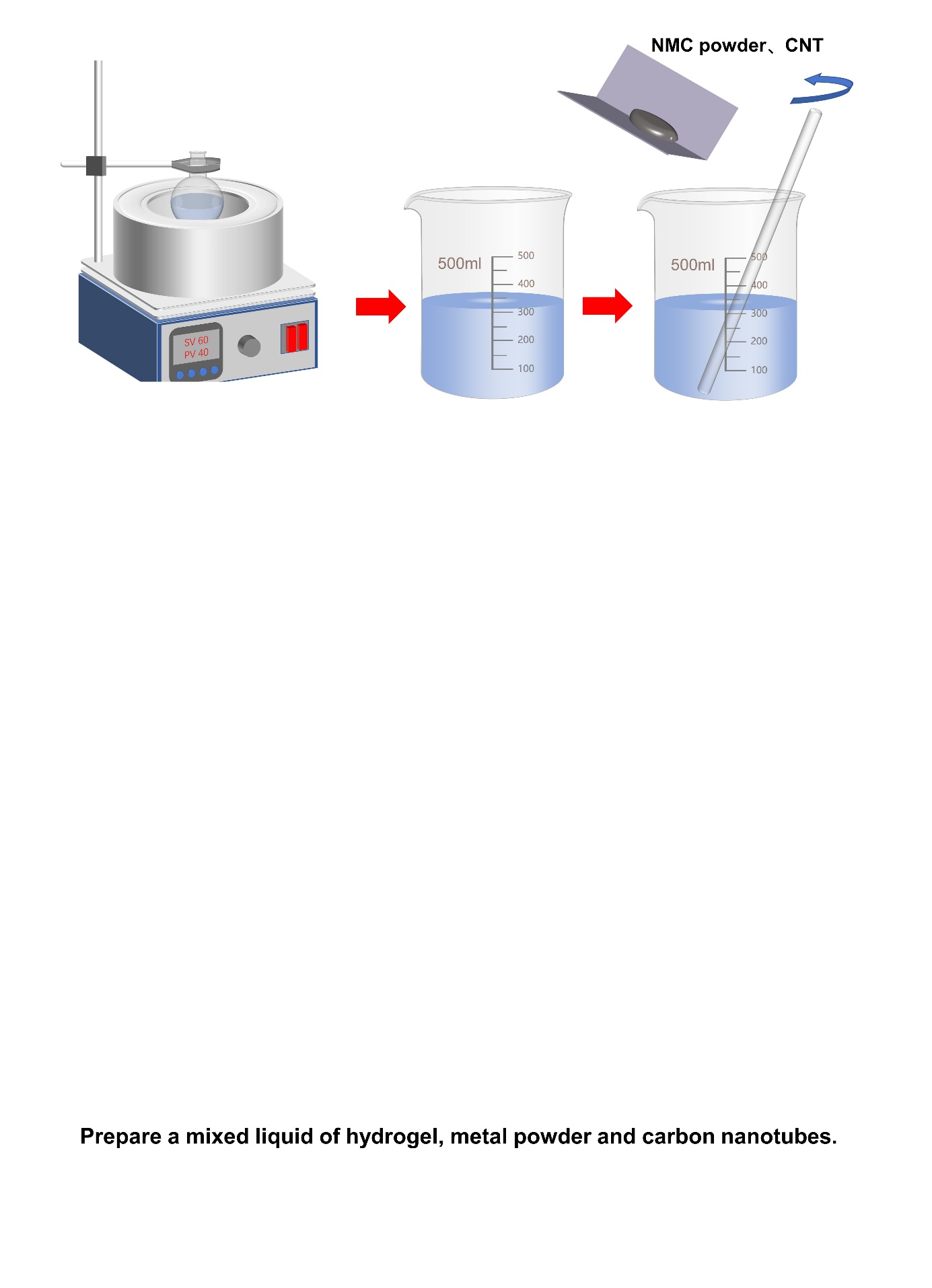


**Fig. S2**. Prepare a mixed liquid of hydrogel, metal powder and carbon nanotubes.


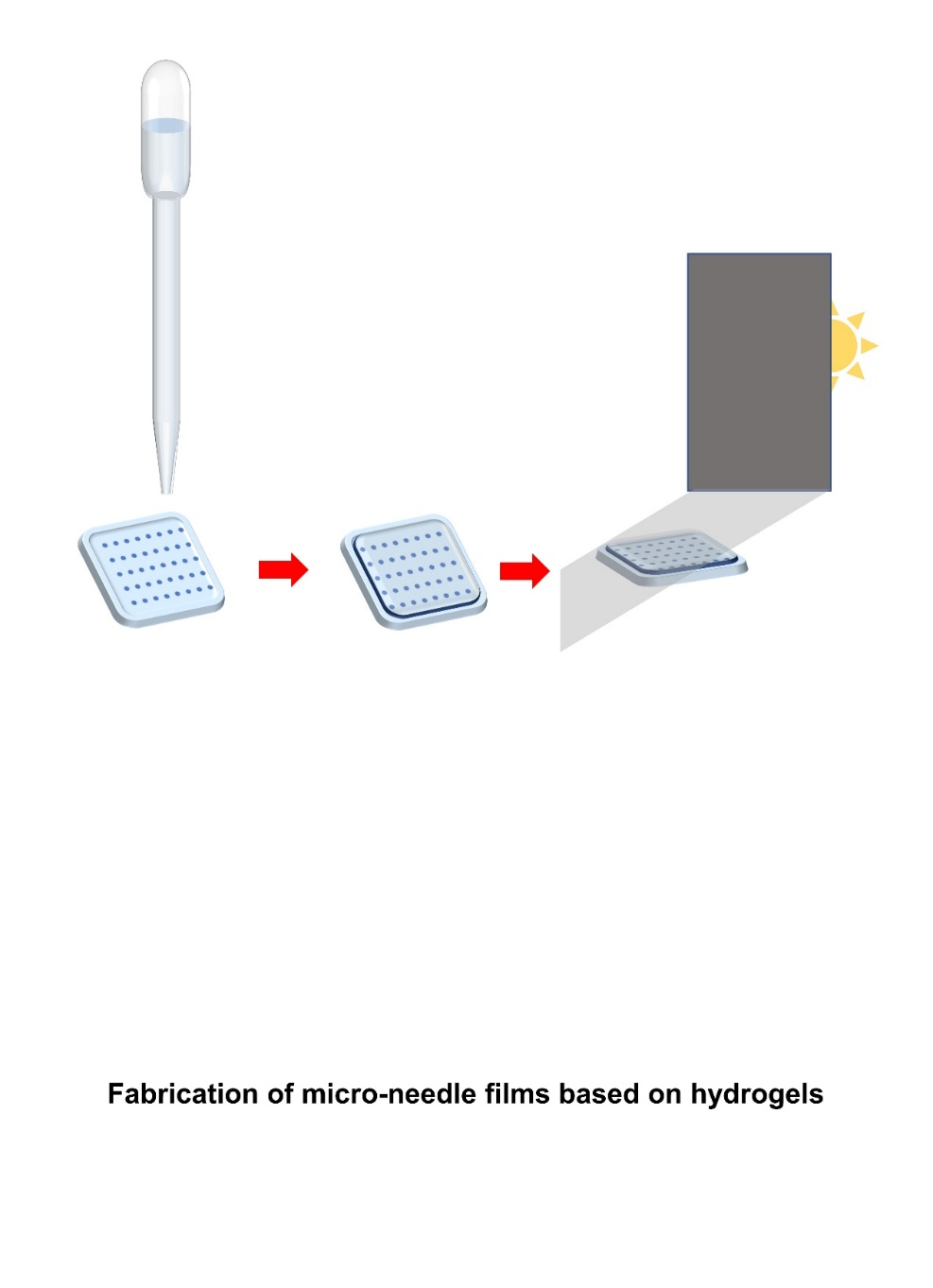


**Fig. S3**. Fabrication of micro-needle films based on hydrogels.

**
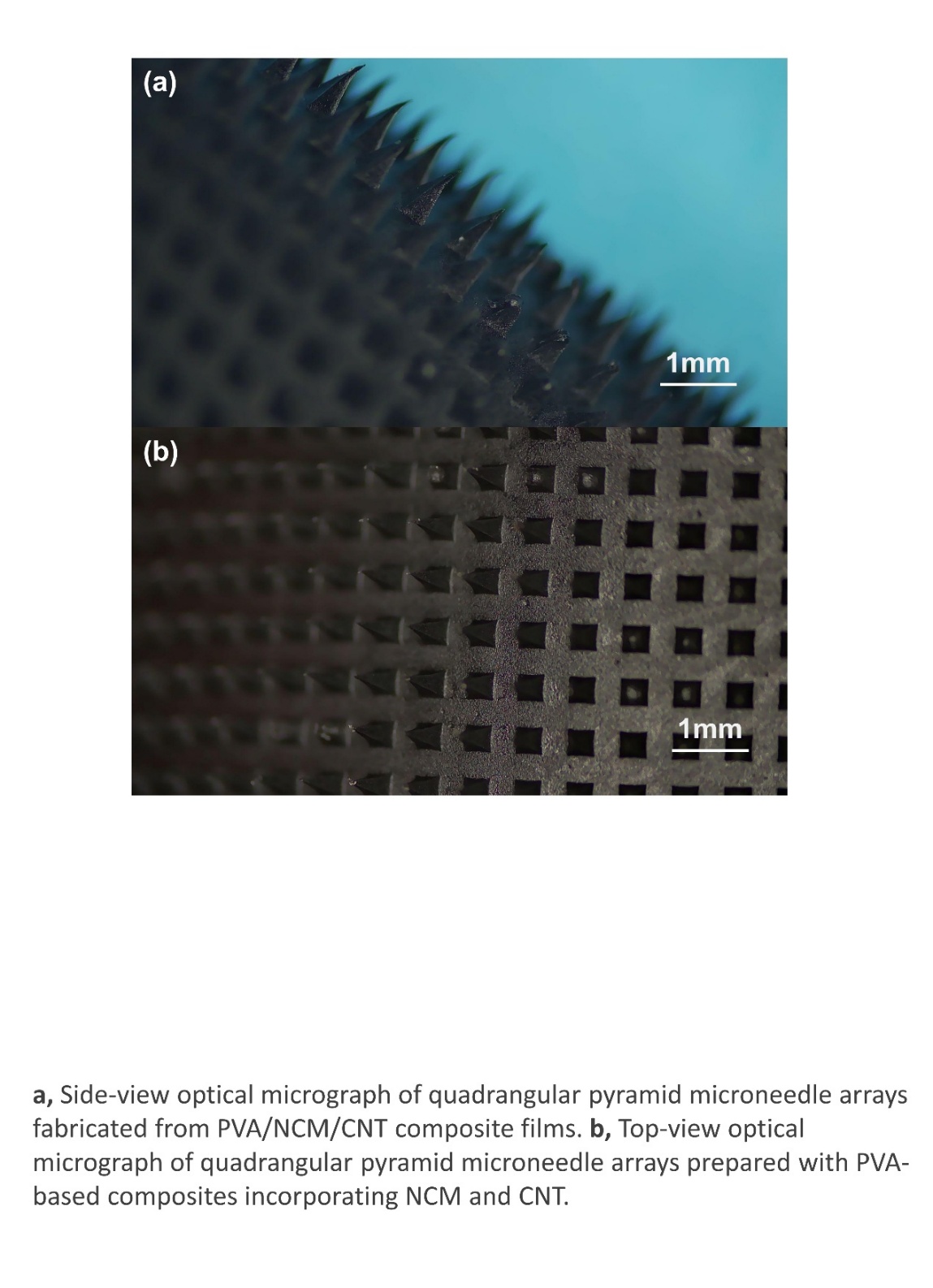
**

**Fig S4.** a) Side-view optical micrograph of quadrangular pyramid microneedle arrays fabricated from PVA/NCM/CNT composite films. b) Top-view optical micrograph of quadrangular pyramid microneedle arrays prepared with PVA-based composites incorporating NCM and CNT.

- 1. ***Test method of electrical performance***

The experiments were performed under atmospheric pressure (1 atm = 101.325 kPa). The electrical output characteristics of the TENGs, including both lithium-ion battery-mimetic architecture TENG (MLBA-TENG) and normal TENG (N-TENG) configurations, were comprehensively evaluated using specialized instrumentation: the output charge was quantified with a Keithley 6517b electrometer, the output voltage was measured with a Oscilloscope, while the output current was similarly determined using the Keithley 6517b electrometer.

# Electrical Performance Analysis of MLBA-TENG

- 1. ***NCM Concentration Experiment***

This study proposes a multifunctional composite material fabricated by incorporating lithium nickel cobalt manganese oxide (NCM) and carbon nanotubes (CNTs) into a polyvinyl alcohol (PVA) matrix. In triboelectric nanogenerator (TENG) applications, the introduction of NCM particles significantly enhances electron transfer efficiency, while the CNT network not only improves the mechanical properties of the material but also optimizes charge collection capability.

To systematically investigate the effect of NCM concentration in the PVA matrix on the electrical output performance of lithium-ion battery-mimetic architecture TENG (MLBA-TENG), four different NCM concentrations were evaluated under identical mechanical energy input conditions. The open-circuit voltage (V_oc_), short-circuit current (I_sc_), and transferred charge (Q_tr_) were measured for each concentration, with 100 datasets collected per condition. The resulting performance distributions are presented in **Fig. S5(a-c)**.


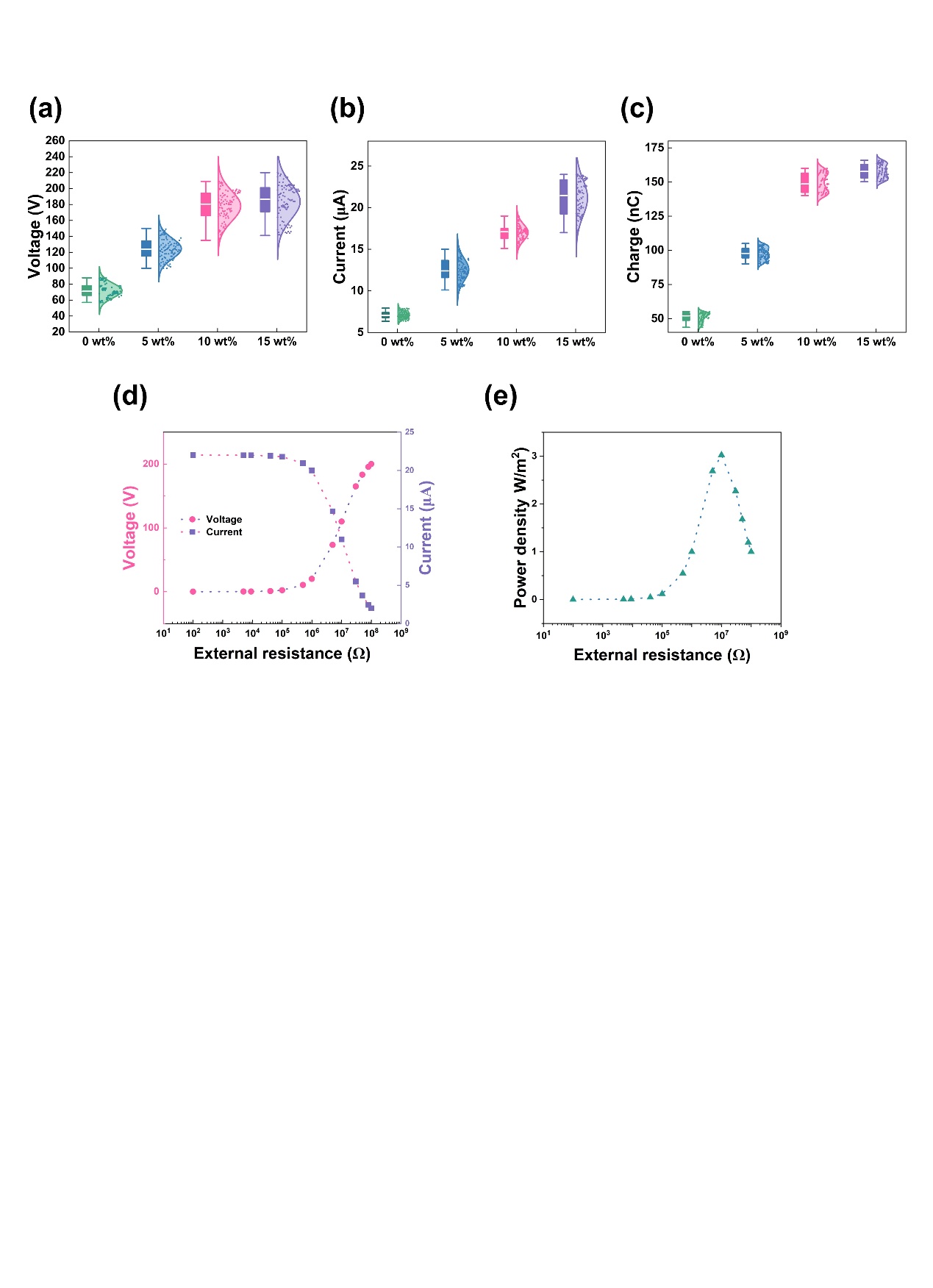


**Supplementary Fig 5.** Electrical performance characterization of MLBA-TENG: (a-c) Output parameter distributions (open-circuit voltage, short-circuit current, and transferred charge) for PVA composites with different NCM concentrations (0, 5, 10, and 15 wt%).

# *Output Performance Analysis of MLBA-TENG Under Load.*

The output characteristics of the TENG exhibit significant dependence on external load resistance (10-1000 MΩ), as shown in **Fig. S6**. With increasing load resistance, the output voltage gradually rises and eventually saturates at 180 V, while the output current continuously decreases, consistent with impedance mismatch theory. In the low-resistance region (<100 MΩ), current-dominated behavior prevails, indicating rapid charge release from the system. Maximum power transfer occurs when the load resistance matches the TENG's internal impedance (approximately 10 MΩ), yielding a peak power density of 2.36 W m^-2^. This optimal matching condition corresponds to the highest practical energy harvesting efficiency.


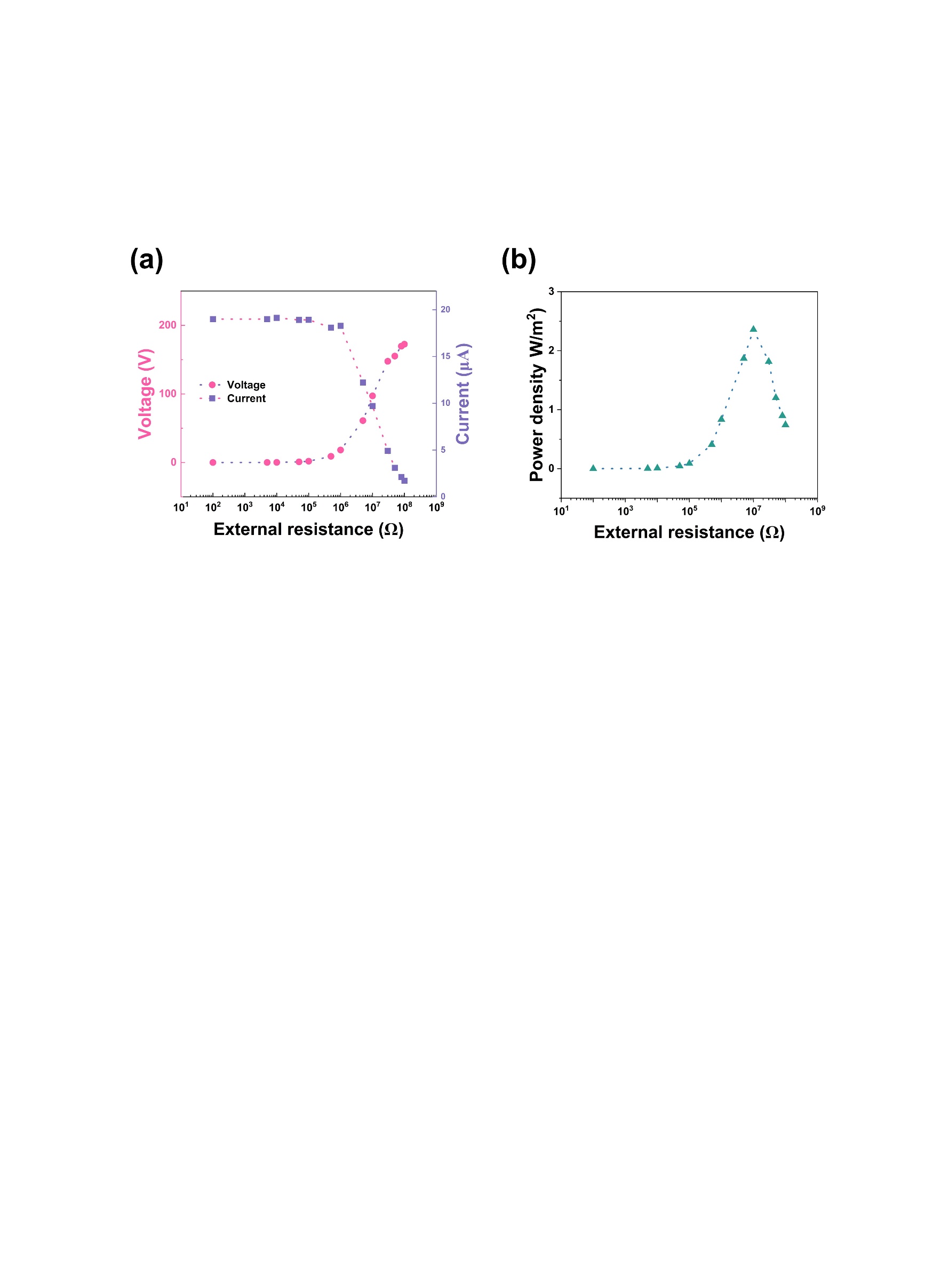


**Fig S6.** a) Current-voltage characteristics under varying load resistances (10-1000 MΩ); b) Output power density of MLBA-TENG under different load conditions.

# Structural Analysis of MLBA-TENG

# *Microneedle Structural Characterization*

The microneedle-structured composite design significantly enhances the triboelectric nanogenerator (TENG) output performance. By constructing well-ordered microneedle arrays, the effective contact area increases by 2.44 times compared to planar films at identical projected dimensions. This architectural advantage directly translates to improved charge generation efficiency. However, such multi-tip configurations introduce new challenges of air breakdown. Under high humidity (>60% RH) or narrow separation gaps (<100 μm), localized electric field concentration occurs at microneedle tips (**Fig. S7**). When the field strength exceeds air's dielectric strength (~3 kV/mm), gas ionization discharge manifests, leading to: (i) accumulated charge leakage through discharge channels, causing measurable output voltage decay, and (ii) potential material degradation through repetitive discharge events^[5]^.


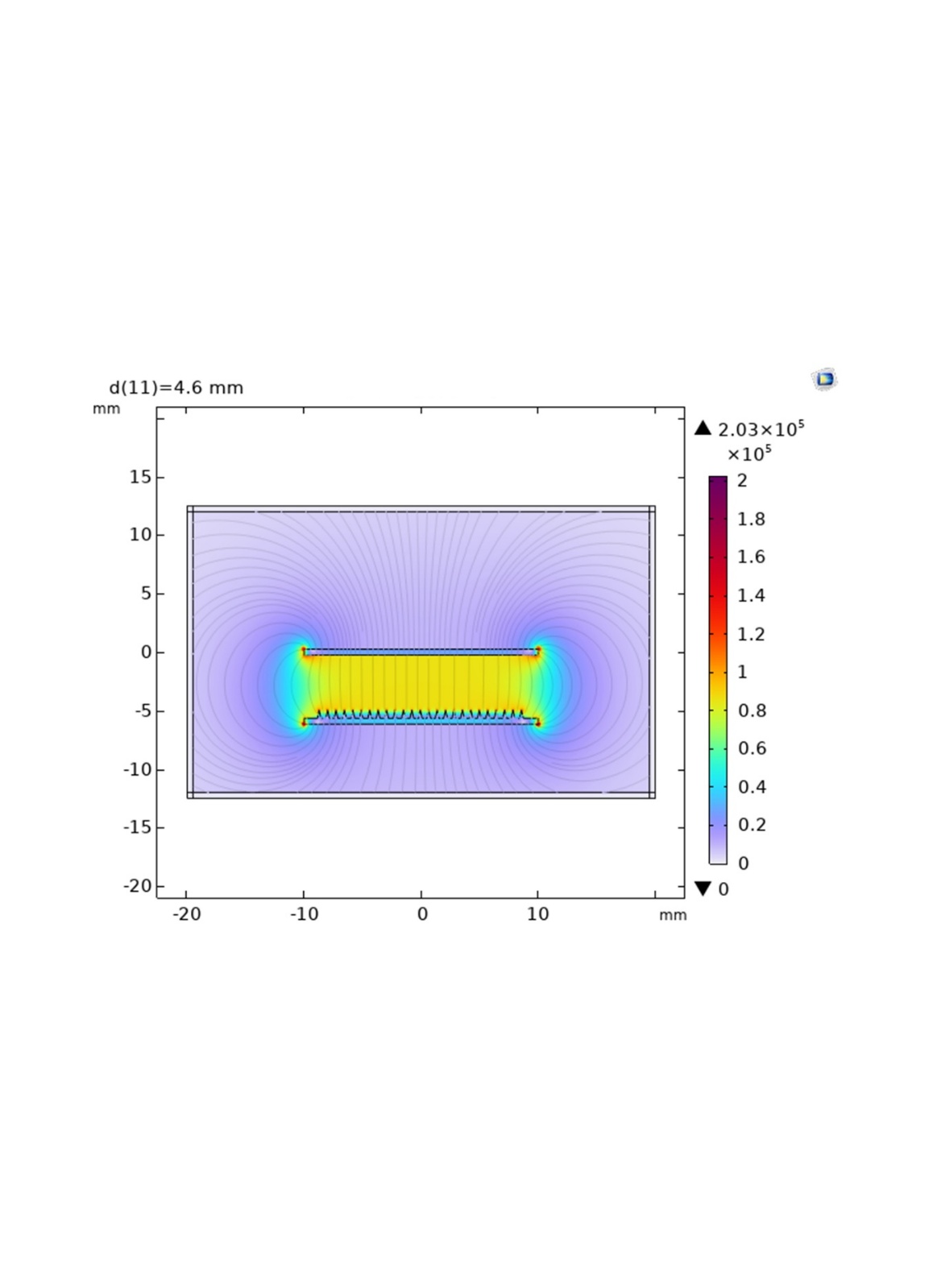


**Fig. S7**. Simulated electric field distribution at a separation distance (d) of 4.6 mm between the triboelectric layers.

# *Composition and Performance Analysis of - mimetic Structures*

The micro-needle array structure of the composite material was analyzed using X-ray energy dispersive spectroscopy (EDS), with **Fig. S9** showing the elemental distribution of a single micro-needle, the micro-needle array, and the nickel-cobalt-manganese oxide elements in the array gaps. Nickel cobalt manganese oxide (NCM) powder was found to be uniformly dispersed throughout the micro-needle membrane, with the mass percentages of each element in the membrane shown in **Fig. S11**. Due to the introduction of the lithium-ion battery-mimetic architecture, the lithium-ion battery-mimetic architecture TENG (MLBA-TENG) exhibited a unique three-peak electrical signal (**Fig. S12**), resulting from the charge transfer mechanism enabled by this structure: as the triboelectric layers separated, the positive charges on the membrane surface were neutralized by electrons released from the oxide metal layer, while the released lithium ions carried positive charges and migrated to the graphite electrode, thereby suppressing voltage release and creating the distinctive three-peak electrical signal. This phenomenon confirms that the lithium-ion battery-like structure effectively suppresses the electric field strength on the membrane surface, reducing the occurrence of air breakdown. The EDS results demonstrate the successful integration of NCM materials into the micro-needle array structure, while the electrical output characteristics reveal the synergistic effect between the triboelectric and electrochemical processes, providing a new approach to enhance TENG performance and stability through material-structure co-design.

Previous studies have achieved breakthrough innovations in the field of TENGs. Specifically, the fully recycled multi-material crumpled-ball TENG (FRMC-TENG) is centered on fully recycled materials, featuring a crumpled-ball design that enables contamination resistance and rapid fabrication, with a high peak current of 1.76 A.^[6]^ The direct-current TENG (DC-TENG) proposes scientific material selection rules; when polyvinyl chloride (PVC) is used as the triboelectric layer, it achieves a record-high effective charge density of 8.80 mC m-2, and its direct-current output can directly drive electronic devices.^[7]^ The opposite-charge-enhanced transistor-like TENG (OCT-TENG) leverages the opposite-charge synergy effect and a transistor-like structure, delivering an ultrahigh instantaneous power density exceeding 10 MW m-2, which is capable of lighting 180 W commercial lamps.^[8]^ However, none of these three TENGs have specifically addressed the electrostatic breakdown issue. In contrast, the mimosa-inspired microneedle-array TENG with a lithium-ion battery-mimetic architecture perfectly resolves this critical challenge through a spontaneous charge storage-release mechanism enabled by its lithium-ion battery-mimetic architecture. This mechanism dynamically suppresses air breakdown, eliminating concerns about charge escape induced by electrostatic breakdown. By integrating a mimosa-inspired microneedle array with a vertical multi-layer design, the MLBA-TENG achieves both high charge density and excellent flexibility, making it well-suited for wearable applications.

In addition, a comparison of the transferred charge density between the MLBA-TENG and TENGs reported in previous studies is provided in **Fig. S14**. Comparative analysis clearly indicates that the proposed device exhibits a performance improvement of several tens of μC/m² or even several times in terms of transferred charge density. This enhancement is primarily attributed to its unique biomimetic lithium-ion architecture and the built-in self-regulating charge compensation mechanism.

In the field of wearable textile-based TENGs, numerous scholars have carried out innovative and in-depth research, laying a solid foundation for the development and practical application of this technology, and their outstanding work provides important support for this study.

Zhao and Hu et al. have long been engaged in the research of wearable textile-based TENG sensors, and have made remarkable achievements in the integration of TENGs with textile structures. They ingeniously adopted textile industry-compatible technologies to fabricate TENG-based textile pressure sensors, which adopted knitting, weaving and embroidery structures with excellent machine washability and high air permeability. On this basis, they further explored and revealed the intrinsic correlation between textile structures and sensor performance, providing valuable theoretical guidance and technical reference for the design and optimization of wearable TENG sensors. Their pioneering work not only enriches the research system of wearable TENGs, but also fully demonstrates the broad application prospects of TENGs in the field of wearable flexible sensors, laying a solid foundation for the practical application of wearable TENG sensors.^[17]^

Hu and Zheng et al. have made systematic and comprehensive achievements in the review and summary of textile-based TENGs (t-TENGs). They comprehensively combed the construction methods of t-TENGs, including three dominant device structures and emerging three-dimensional textile-based design schemes, and systematically reviewed the influence rules of textile processing methods (weaving, knitting, sewing, etc.) and structural patterns on the performance of t-TENGs. In addition, they also demonstrated the excellent washability and tailorability of t-TENGs through optimized material selection and rational device design, which solved the key practical problems that have long restricted the development of wearable electronic textiles. Finally, they prospected the development trend of t-TENGs, focusing on the integration of t-TENGs with other energy harvesting and storage technologies, providing important guidance for the follow-up research direction of t-TENGs and effectively promoting the sustainable development of wearable electronic textiles.^[18]^

Xu and Yu et al. have made important breakthroughs in the field of wearable haptic human-machine interfaces (HMI) based on TENGs, effectively solving the technical bottlenecks of traditional haptic devices. Aiming at the defects of traditional haptic devices such as bulkiness, poor comfort and poor portability caused by cumbersome instruments and power modules, they innovatively proposed a thin, soft and self-powered electrotactile textile haptic (SPETH) glove. The glove realizes localized electrical stimulation by utilizing the triboelectric effect and gas breakdown discharge, and can harvest mechanical energy from daily hand movements to achieve self-power supply, without relying on external power equipment. With the advantages of softness, light weight, self-sustainability, portability and low cost, their designed SPETH glove breaks through the technical limitations of traditional haptic devices, provides a new technical path for the development of battery-free and high-comfort wearable HMI, and shows great application potential in virtual reality/augmented reality, prosthetic control, therapeutic applications and other fields.^[19]^

Ali Iftikhar, Karim Nazmul, Afroj Shaila and their colleagues have made remarkable achievements in the research of high-performance self-powered wearable electronic systems, and have made important progress in the integration of TENG energy harvesting and storage. They innovatively integrated a textile-based TENG (T-TENG) based on 2D materials with a textile supercapacitor (T-Supercap) into cotton fabric, successfully realizing the integration of energy harvesting and storage in wearable textiles. By introducing 2D materials (graphene and MoS2) into the fabrication process, they significantly improved the electrical performance of T-TENG, achieving a record-high open-circuit voltage of 1068 V and a power density of 14.64 W/m² under a force of 22 N. The T-TENG developed by them has excellent practical performance, which can effectively power more than 200 LEDs and a miniature watch, and can charge T-Supercap with a force of 4-5 N to ensure the stable operation of miniature electronic devices. In addition, they integrated the T-TENG into socks as a self-powered step counter sensor, further expanding the application scenario of wearable TENGs. Their work establishes a high-performance, stable and reliable platform for wearable electronic textiles, making an important contribution to the advancement of sustainable and autonomous self-powered wearable technologies.^[20]^

The research team of the article titled Thin, soft, garment-integrated triboelectric nanogenerators for energy harvesting and human machine interfaces has made important progress in the research of garment-integrated TENGs (G-TENGs), providing important technical support for the deep integration of TENGs with wearable garments. They systematically reported the material selection, device design scheme and processing route of G-TENGs, and fully demonstrated the application potential of G-TENGs in wearable energy harvesting and human-machine interfaces. The G-TENGs designed by them adopt a simple and reliable structure, including two soft silicone layers and one graphene-coated fabric layer, which not only has excellent flexibility, air permeability and robust durability, but also exhibits outstanding electrical performance. Under the conditions of constant frequency of 3 Hz and stress of 5.6 kPa, the open-circuit voltage and short-circuit current of G-TENGs can reach 213.75 V and 3.11 μA respectively. Moreover, the G-TENGs can maintain stable electrical output after more than 1000 cycles of bending, stretching and twisting, showing excellent mechanical stability and service life. Their research effectively promotes the integration of TENGs with garments, and provides important technical support for the practical application of TENGs in wearable electronics.^[21]^ To verify the durability of the MLBA-TENG, a 10,000-cycle continuous stability test was performed under constant pressure and frequency. After long‑term cycling, SEM characterization was carried out to examine the morphological evolution of the microneedle array on the tribopositive layer. The SEM images reveal that slight surface wear occurs on the microneedle structures after repeated mechanical impact, while no obvious fracture or structural collapse is observed **(Fig. S10)**. These results confirm that the MLBA-TENG possesses reliable structural robustness and excellent long‑term operational durability.


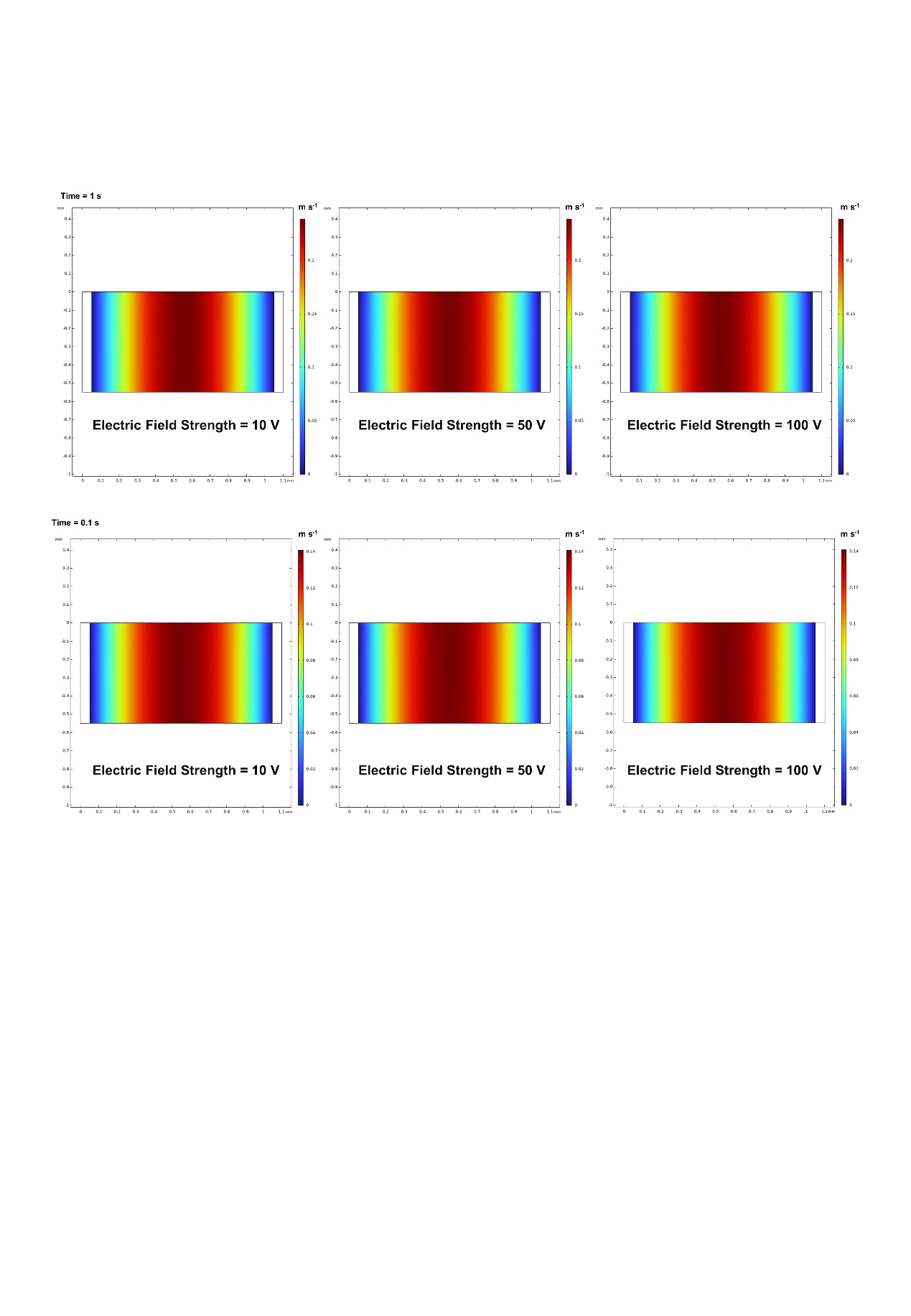


**Fig. S8**. The effect of electric field strength on the drift velocity of lithium ions in the lithium battery- mimetic architecture.


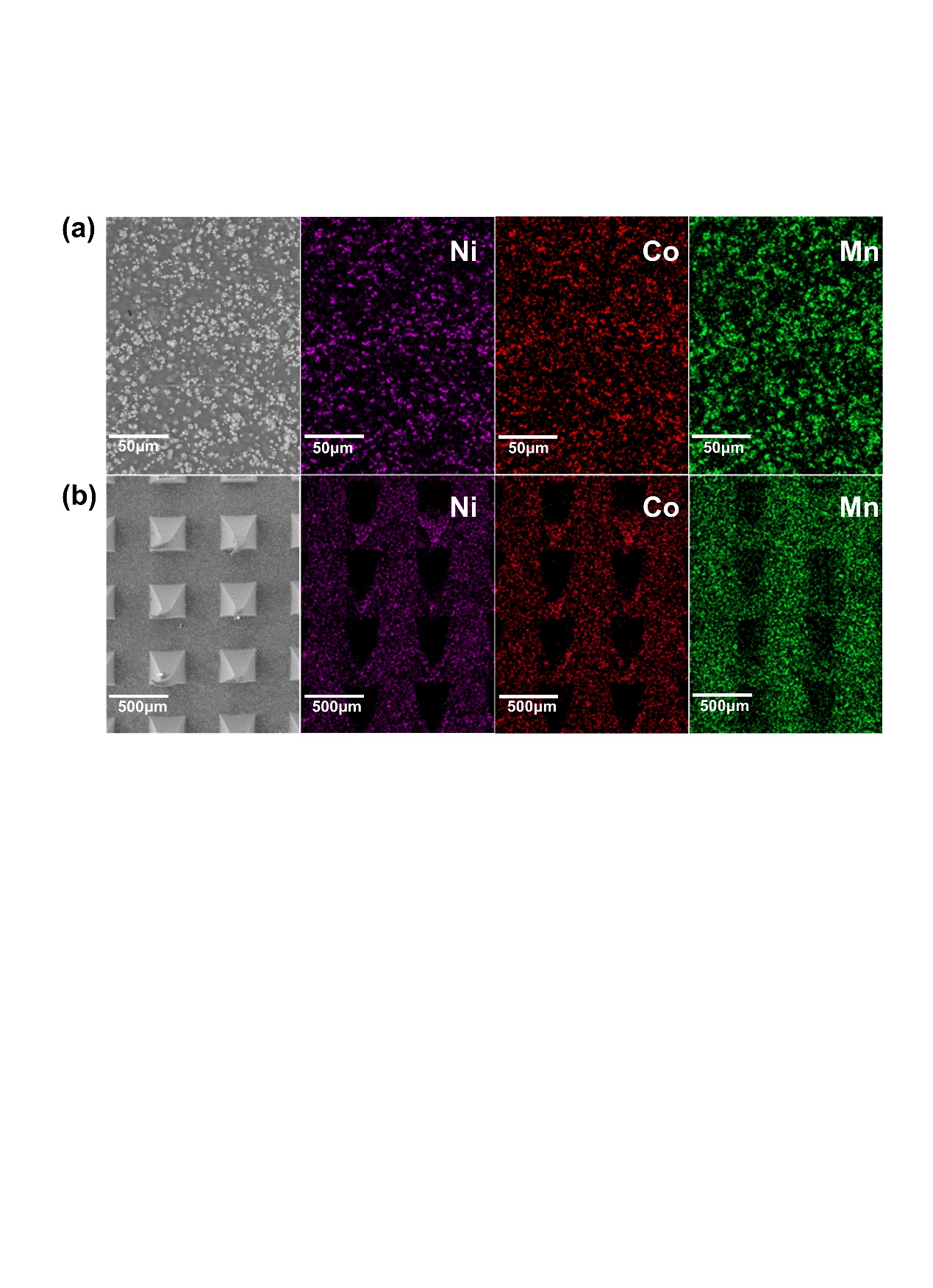


**Fig. S9.** a) Morphological analysis and structural characterization of the material. SEM image of a microneedle structure in the NCM/CNT/PVA composite, where pink, red, and green colors correspond to nickel (Ni), cobalt (Co), and manganese (Mn) elements, respectively. b) SEM image showing the inter-needle spacing within the NCM/CNT/PVA composite microneedle array.





**Fig. S10.** SEM image of the microneedle structure after 10,000-cycle stability test.


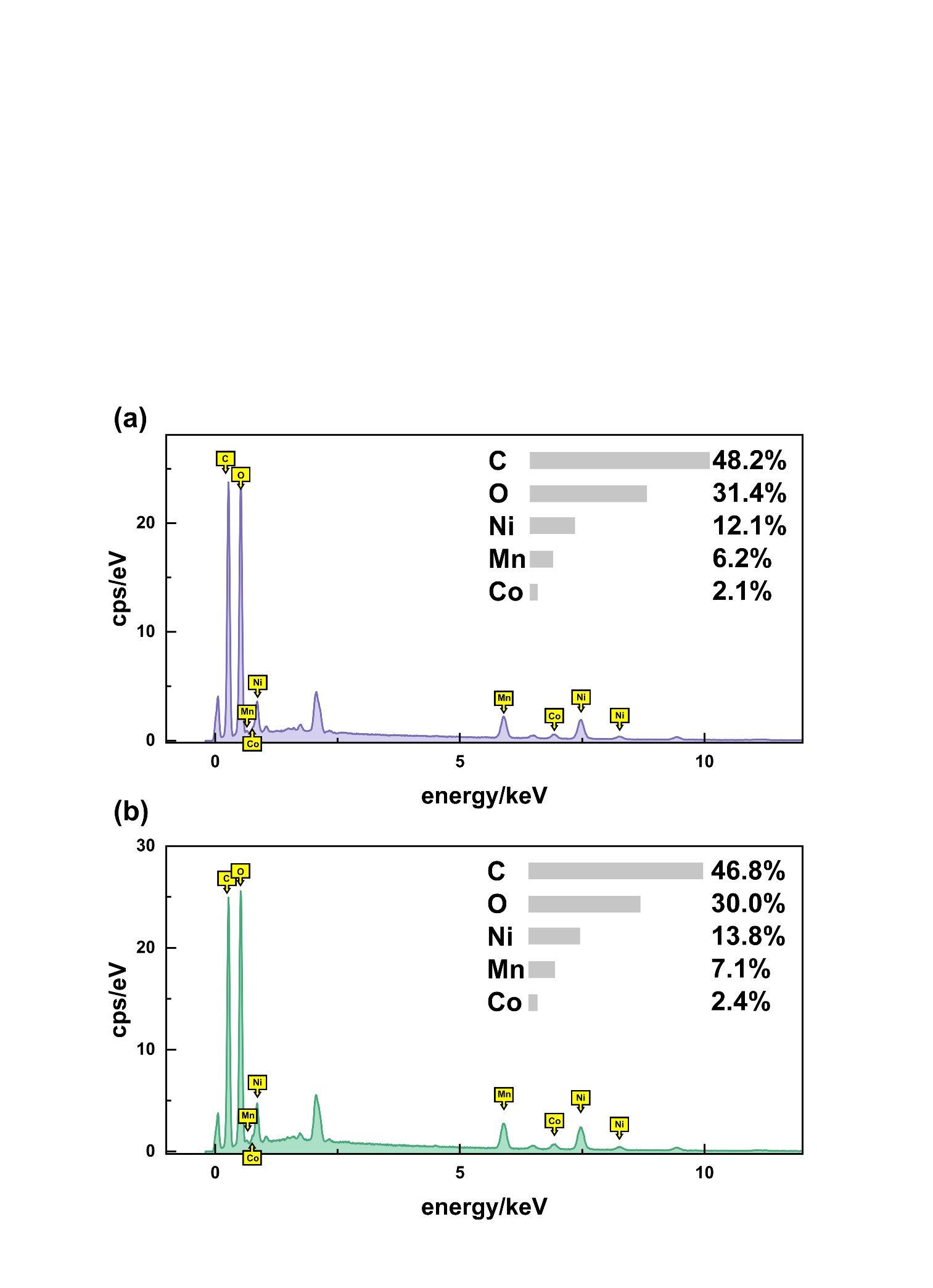


**Fig. S11**. a, b) The EDS spectra (a, b) of the composite material correspond to the structures shown in Fig. S9.


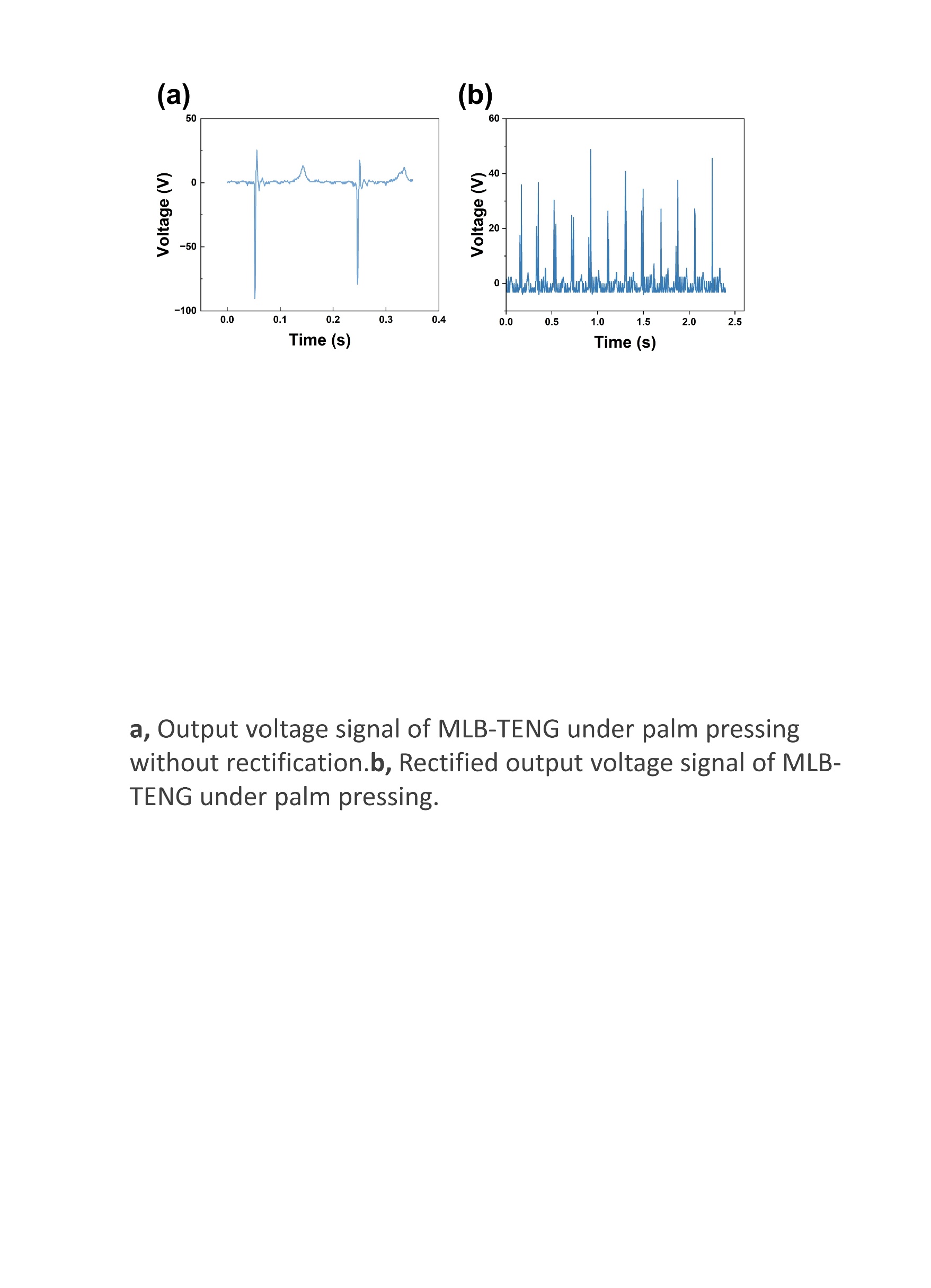


**Fig S12.** a) Output voltage signal of MLBA-TENG under palm pressing without rectification. b) Rectified output voltage signal of MLBA-TENG under palm pressing.


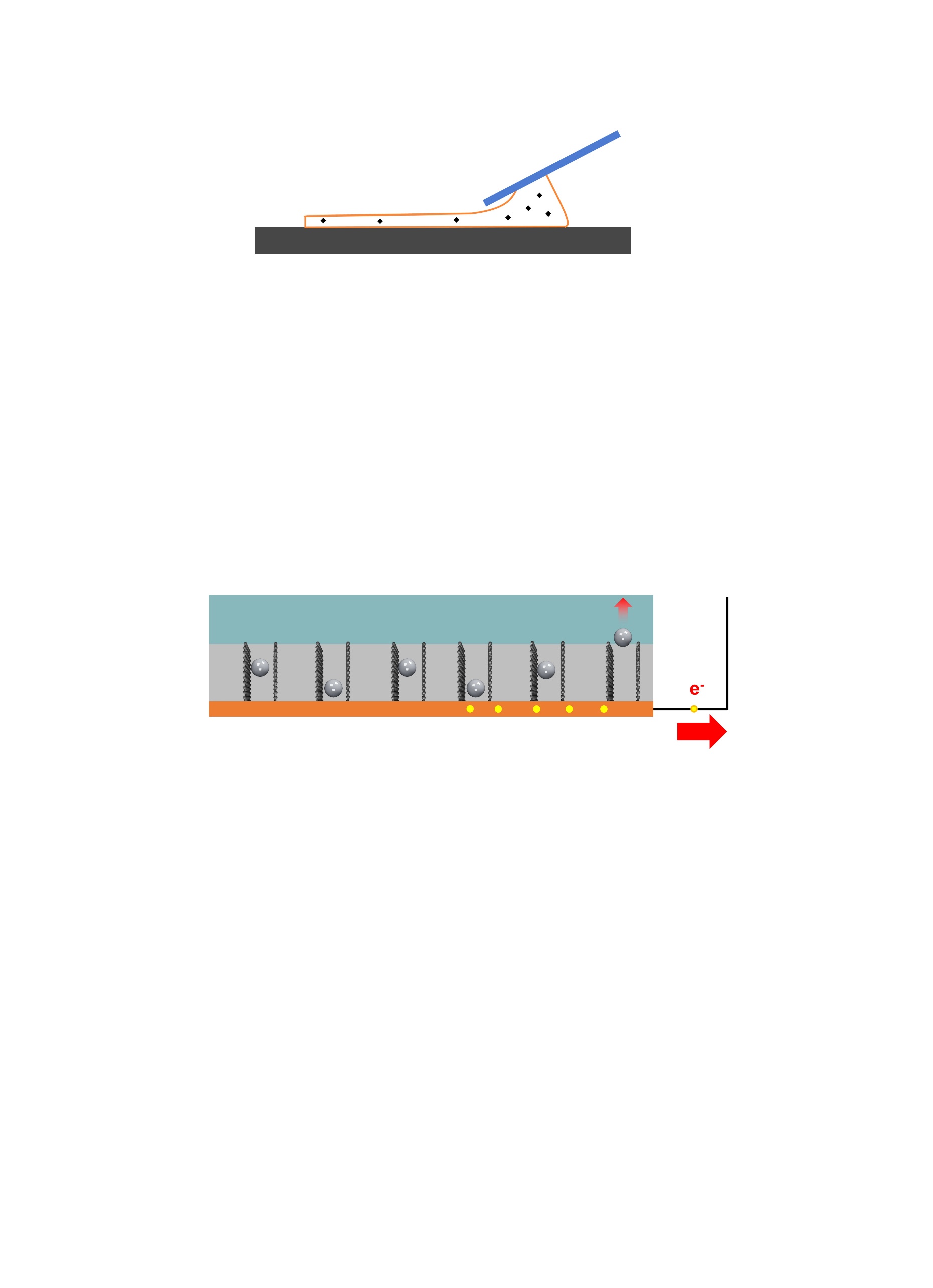


**Fig. S13**. Lithium-ion deintercalation from graphite electrode.


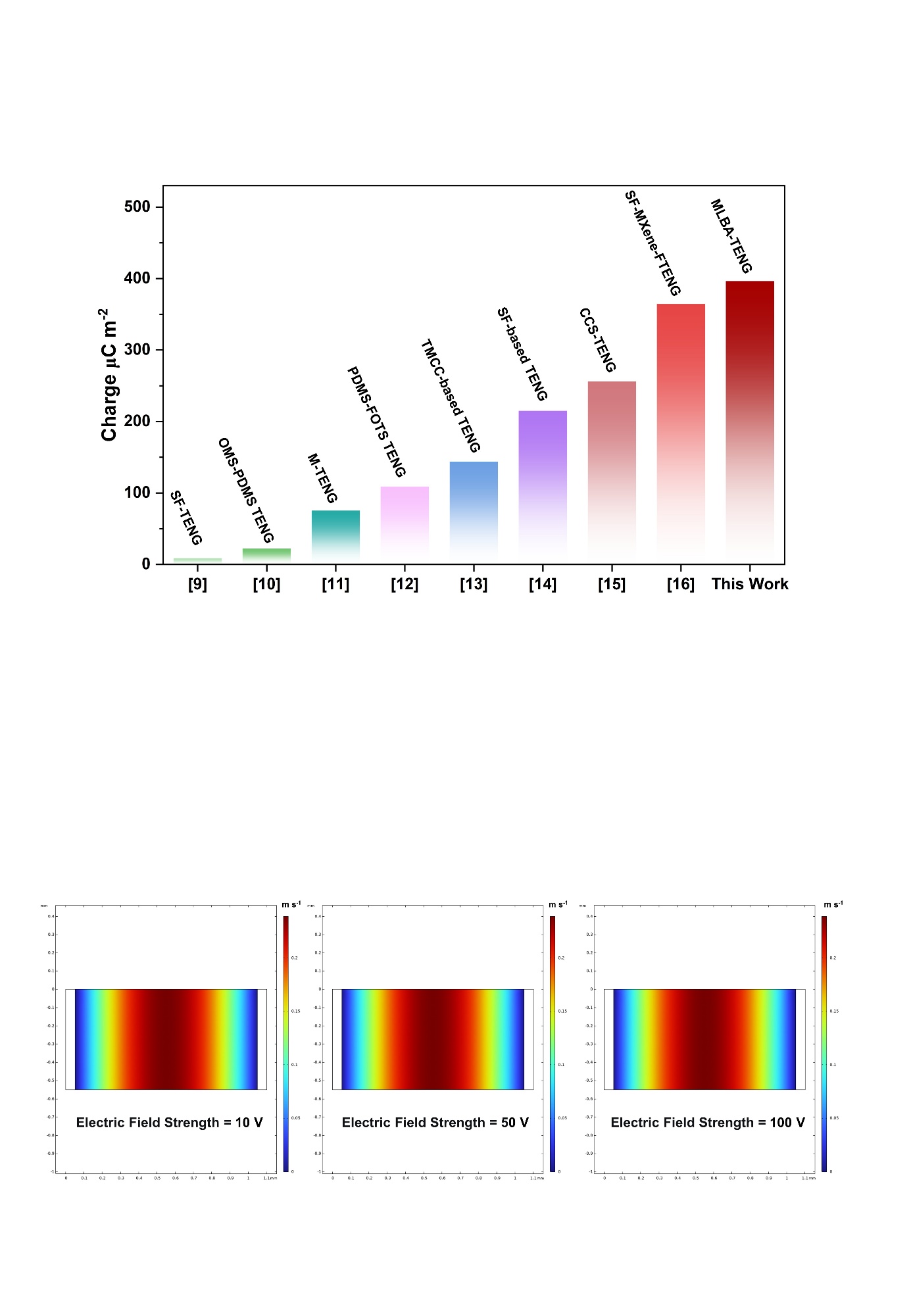


**Fig. S14**. Comparison of transferred charge density between MLBA-TENG and other TENGs in previous studies.


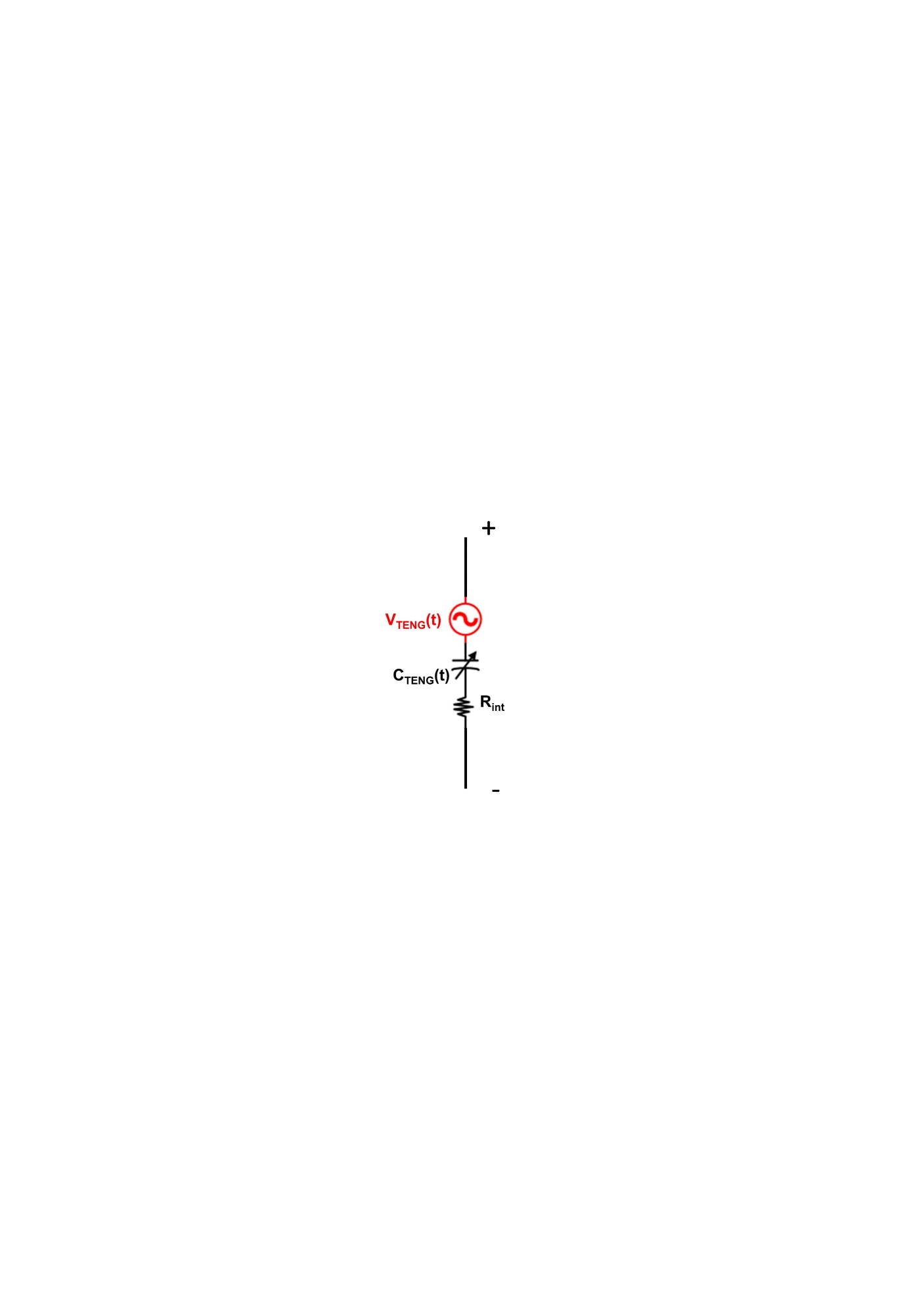


**Fig. S15.** Equivalent circuit diagram of conventional TENGs.


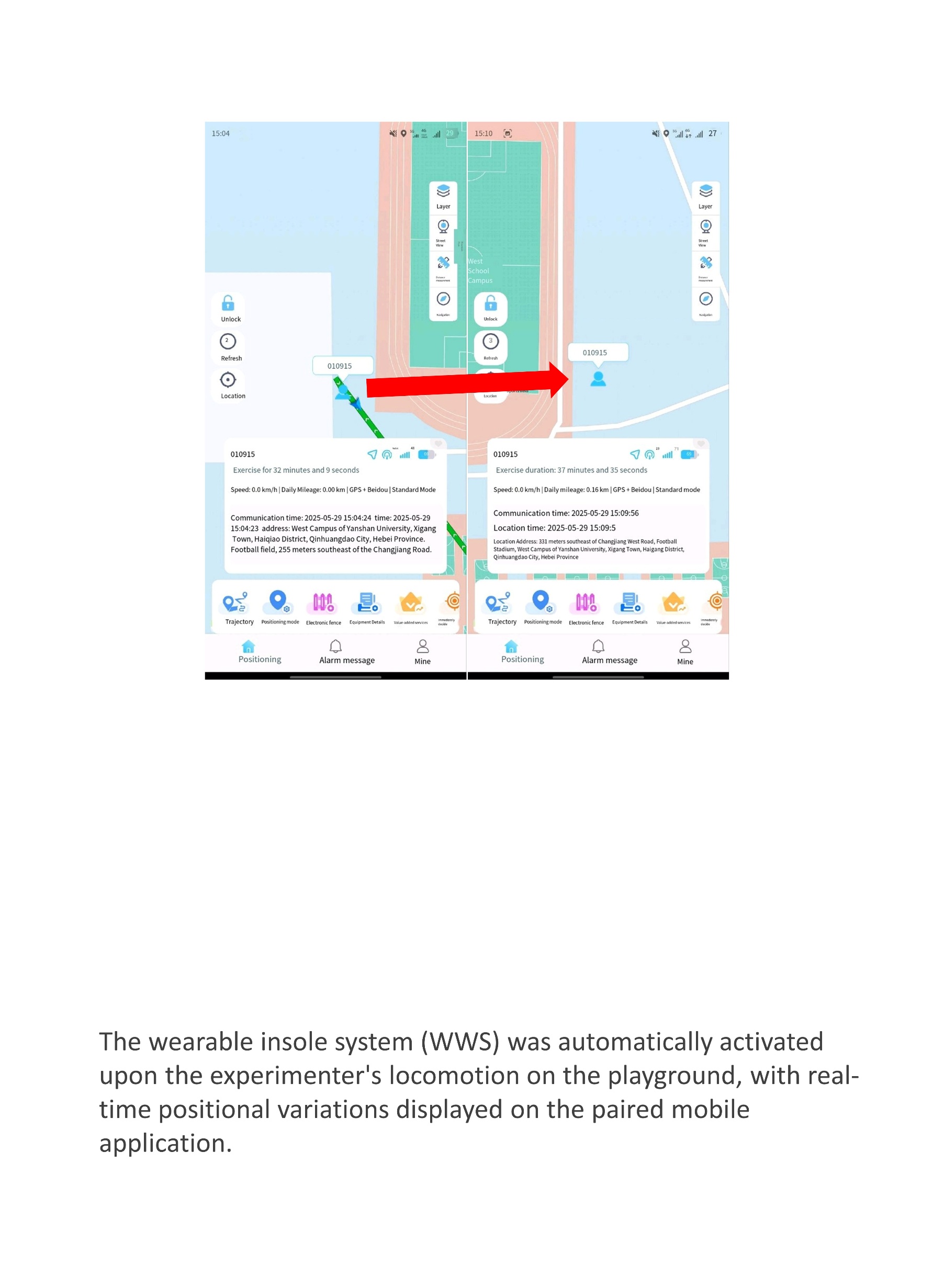


**Fig. S16**. The wearable insole system was automatically activated upon the experimenter's locomotion on the playground, with real-time positional variations displayed on the paired mobile application.

# Electrochemical Simulation and Analysis

# *Model Formulation*

The electrochemical behavior of the proposed polyvinyl alcohol (PVA) based lithium-ion battery is described using a Newman-type porous electrode model, which couples charge conservation and mass transport in both solid and electrolyte phases. The governing equations are formulated as follows:

1. Solid-Phase Charge Conservation

For the anode (nickel cobalt manganese oxide (NCM)/PVA/ carbon nanotubes (CNT) composite):

$$\begin{aligned} \nabla\cdot\left( \sigma_{s,eff}^{a}\nabla\phi_{s}^{a} \right)=-a_{s}j_{int}^{a}\#\left( 1 \right) \end{aligned}$$

Where $\sigma_{s,eff}^{a}=\sigma_{NCM}\cdot\varepsilon_{NCM}^{1.5}\cdot\left( 1-\varepsilon_{PVA} \right)^{1.5}$ is the effective electronic conductivity, $\phi_{s}^{a}$ is the solid-phase potential, $a_{s}$ is the specific interfacial area, $j_{\text{int}}^{a}$ is the interfacial current density. The parameters *σ_NCM_*, *ε_NCM_*, and *ε_PVA_* correspond to the intrinsic electronic conductivity of the NCM material, the volume fraction of NCM active material, and the volume fraction of the PVA polymer matrix respectively.

For the cathode (graphite):

$$\begin{aligned} \nabla\cdot\left( \sigma_{s,eff}^{c}\nabla\phi_{s}^{c} \right)=-a_{s}j_{int}^{c}\#\left( 2 \right) \end{aligned}$$

1. Electrolyte Phase Transport

The electrolyte (PVA-LiPF₆) is governed by:

$$\begin{aligned} \frac{\partial\left( \varepsilon_{e}c_{e} \right)}{\partial t}=\nabla\cdot\left( D_{e,eff}\nabla c_{e} \right)+\frac{1-t_{+}^{0}}{F}a_{s}j_{int}\#\left( 3 \right) \end{aligned}$$

Where the effective diffusion coefficient $D_{e,eff}=D_{e}\cdot\varepsilon_{e}^{1.5}$ accounts for the tortuous PVA matrix, $t_{+}^{0}$ is the Li⁺ transference number, $c_{e}$ is the electrolyte Li⁺ concentration, $\varepsilon_{e}$ is the porosity (volume fraction of voids) in the electrolyte.

Charge conservation in the electrolyte is given by:

$$\begin{aligned} \nabla\cdot\left( \kappa_{eff}\nabla\phi_{e} \right)+\nabla\cdot\left( \frac{2\kappa_{eff}RT}{F}\left( 1-t_{+}^{0} \right)\nabla\ln c_{e} \right)=-a_{s}j_{int}\#\left( 4 \right) \end{aligned}$$

where $\kappa_{\text{eff}}$ is effective ionic conductivity which is reduced due to the inherently low ionic conductivity of the PVA polymer matrix. The parameters R, T, and F correspond to the universal gas constant, temperature, and Faraday's constant respectively, where R = 8.314 J/(mol·K) represents the fundamental thermodynamic constant relating energy to molar quantities, T denotes the absolute temperature in Kelvin (typically 298 K for standard conditions), and F = 96,485 C/mol serves as the critical conversion factor between molar quantities and electrical charge in electrochemical systems.

# *Interfacial Reaction Kinetics (Butler-Volmer Equation)*

The charge-transfer reaction at the electrode-electrolyte interface follows:

$$\begin{aligned} j_{\text{int}}=j_{0}\left[ \exp\left( \frac{\alpha_{a}F\eta}{\mathrm{RT}} \right)-\exp\left( -\frac{\alpha_{c}F\eta}{\mathrm{RT}} \right) \right]\#\left( 5 \right) \end{aligned}$$

The $j_{0}$ is the exchange current density. The overpotential η is calculated as the difference between the electrode solid-phase potential $\phi_{s}$, the electrolyte liquid-phase potential $\phi_{e}$, and the equilibrium potential *U_eq_* of the electrochemical reaction:

$$\begin{aligned} \eta=\phi_{s}-\phi_{e}-U_{eq}\#\left( 6 \right) \end{aligned}$$

# *Ohmic Polarization Analysis*

The total cell resistance is dominated by the electrolyte:

$$\begin{aligned} R_{total}=\frac{\delta_{a}}{\sigma_{s,eff}^{a}}+\frac{\delta_{e}}{\kappa_{eff}}+\frac{\delta_{c}}{\sigma_{s,eff}^{c}}\#\left( 7 \right) \end{aligned}$$

# *Concentration Polarization Effects*

At 1C discharge, the electrolyte concentration gradient leads to:

$$\begin{aligned} \Delta c_{e}\approx\frac{I\delta_{e}}{2FD_{e,eff}}\#\left( 8 \right) \end{aligned}$$

This causes a voltage drop of:

$$\begin{aligned} \Delta V_{conc}=\frac{2RT}{F}\left( 1-t_{+}^{0} \right)\ln\left( \frac{c_{e,0}+\Delta c_{e}}{c_{e,0}} \right)\#\left( 9 \right) \end{aligned}$$

# *Rate Performance Limitations*

The limiting current density due to solid-phase diffusion is:

$$\begin{aligned} I_{lim}=\frac{3FD_{s}^{a}c_{s,max}}{R_{p}}\#\left( 10 \right) \end{aligned}$$

where $R_{p}$​ is the NCM particle radius.This suggests that at high C-rates (>0.5C), the anode becomes diffusion-limited, reducing usable capacity.

# *Potential Optimization Strategies*

To enhance the electrochemical performance of the PVA-based composite battery, several optimization strategies can be implemented. First, the electrolyte modification should be considered to improve ionic conductivity, such as adding plasticizers to increase $\kappa_{\text{eff}}$​ to ~0.5 S/m or incorporating ceramic fillers to further enhance Li⁺ transport. Second, the anode structure requires optimization by increasing the NCM wt% from 10% to 15% to improve capacity, though this necessitates better CNT percolation to maintain electronic conductivity ^[22]^. Additionally, reducing the NCM particle size ($R_{p}$​<1μm) would significantly enhance solid-state diffusion kinetics. Third, interfacial engineering plays a crucial role in reducing charge-transfer resistance, which can be achieved through surface coating of NCM particles and increasing the CNT content to establish a more robust conductive network while preserving mechanical stability. These modifications collectively target the key limitations identified in the model—low ionic conductivity, slow solid-state diffusion, and insufficient percolation—and are expected to improve both energy and power density substantially.

# Performance Simulation

In the present work, a simulation model was established using COMSOL software to simulate the electrical properties of the friction layer and the electrochemical performance of the lithium-ion battery-like structure, with the simulation parameters listed in Table S1 and Table S2.

**Table. S1** Simulation parameters of the triboelectric nanogenerator (TENG)

| Parameter | Value | Description |
| --- | --- | --- |
| d | 5 mm | Thickness of the friction layer |
| a | 0.72 mm | Height of the microneedles |
| fp1 | 0 | Floating potential of the bottom electrode |
| fp2 | 0 | Floating potential of the top electrode |
| ρs1 | -1.00×10⁻⁶ C/m² | Surface charge density of the PDMS layer |
| ρs2 | 4.24×10⁻⁷ C/m² | Surface charge density of the PVA layer |
| V0 | 0 V | Potential at infinity |
| r1 | 20 mm | Width of the friction layer |
| r2 | 0.5 mm | Height of the friction layer |
| v | 0.5 mm/s | Separation velocity |

**Table. S2** Electrochemical simulation parameters of the lithium-ion-battery-mimetic architecture.

| Parameter | Value | Description |
| --- | --- | --- |
| i_1C | 17 A/m² | 1C discharge current density |
| Ds_neg | 3.9×10⁻¹⁴ m²/s | Solid-phase diffusion coefficient (anode) |
| Ds_pos | 1×10⁻¹³ m²/s | Solid-phase diffusion coefficient (cathode) |
| rp_neg | 12.5 μm | Anode particle radius |
| rp_pos | 8 μm | Cathode particle radius |
| T | 298 K | Temperature |
| epsl_pos | 0.63 | Electrolyte phase volume fraction (cathode) |
| Epss_pos | 1-epsl_pos-epss_filler_pos | Active material volume fraction (cathode) |
| epss_filler_pos | 0.073 | Conductive filler fraction (cathode) |
| cl_0 | 700 mol/m³ | Initial electrolyte salt concentration |
| epsl_neg | 0.503 | Electrolyte phase volume fraction (anode) |
| epss_filler_neg | 0.026 | Conductive filler fraction (anode) |
| epss_neg | 1-epsl_neg-epss_filler_neg | Active material volume fraction (anode) |
| csmax_neg | 26,390 mol/m³ | Max. solid-phase concentration (anode) |
| csmax_pos | 22,860 mol/m³ | Max. solid-phase concentration (cathode) |
| cs0_neg | 14,870 mol/m³ | Initial active material concentration (anode) |
| cs0_pos | 3,900 mol/m³ | Initial active material concentration (cathode) |
| ks_neg | 100 S/m | Solid-phase conductivity (anode) |
| ks_pos | 100 S/m | Solid-phase conductivity (cathode) |
| i0_neg_ref | 0.11 mA/cm² | Reference exchange current density (anode) |
| i0_pos_ref | 0.08 mA/cm² | Reference exchange current density (cathode) |
| cl_ref | 700 mol/m³ | Reference electrolyte salt concentration |
| cs_neg_ref | 14,870 mol/m³ | Reference active material concentration (anode) |
| cs_pos_ref | 3,900 mol/m³ | Reference active material concentration (cathode) |

**Table S3 GPS module parameters**

| Parameter | Value |
| --- | --- |
| Size | 30 mm ×30 mm |
| Positioning Accuracy | 10 m |
| Battery Capacity | 400 mAh |
| Positioning Frequency | 0.1 Hz |
| Battery Life (0.1Hz) | 5 h |
| Positioning Interface | UART |
| Communication Module | 2G/4G Cat.1 |

**References**

[1] H. Jeong, H. Jung, M. Dubajic, G. Kim, W. Jeong, H. Song, Y. Lee, S. Biswas, H. Kim, B. Lee, J. Yoon, S. Stranks, S. Jeong, J. Lee, H. Choi, *Nat. Commun.* **2025**, *16* (1), 854.

[2] M. Chen, Y. Zhao, D. Wen, W. Gong, Y. Chen, Y. Gao, Y. Yu, G. Xing, Y. Zhang, W. Zhu, T. Ben, *Nat. Commun.* **2025**, *16* (1), 5499.

[3] J. Wang, Z. Xia, H. Yao, Q. Zhang, H. Yang, *Acs Applied Materials & Interfaces* **2023**, *15*(40) 47208-47220.

[4] H. Kang, T. Park, S. Song, Y. Yoon, S. Lee, *Nanomaterials* **2024**, *14* (13), 1096.

[5] Z. Cao, J. Teng, R. Ding, J. Xu, C. Ren, S. Lee, X. Guo, X. Ye, *Energy Environ. Sci.* **2024**, *17* (14), 5002-5012.

[6] M. Jee, K. Cha, S. Chung, H. Yong, Y. Jin, J. Hong, S. Lee, EcoMat **2025**, 7 (12), e70040.

[7] Z. Zhao, L. Zhou, S. Li, D. Liu, Y. Li, Y. Gao, Y. Liu, Y. Dai, J. Wang, Z. Wang, Nat. Commun. **2021**, 12 (1), 4686.

[8] H. Wu, S. Wang, Z. Wang, Y. Zi, Nat. Commun. **2021**, 12 (1), 5470.

[9] K. Chen, Y. Li, G. Yang, S. Hu, Z. Shi, G. Yang, Adv. Funct. Mater. **2023**, 33 (45), 2304809.

[10] W. Li, Y. Xiang, W. Zhang, K. Loos, Y. Pei, Nano Energy **2023**, 113, 108539.

[11] Y. Park, Y. Ro, Y. Shin, C. Park, S. Na, Y. Chang, H. Ko, Adv. Sci. **2023**, 10 (36), 2304598.

[12] S. Lai, H. Hsu, J. Wu, Nano Energy **2024**, 131, 110158.

[13] Y. Xiao, Z. Li, D. Tan, G. Carsten, B. Xu, Adv. Sci. **2024**, 11 (43), 2409619.

[14] X. Tan, Z. Huang, H. Pei, Z. Jia, J. Zheng, ACS Sens. **2024**, 9 (8), 3938−3946

[15] X. Li, Y. Gao, Y. Hu, L. Lu, Z. Zhao, W. Ma, W. Qiao, X. Liu, Z. Wang, J. Wang, Chem. Eng. J. **2024**, 487, 150449.

[16] X. Tan, Z. Huang, L. Chang, H. Pei, Z. Jia, J. Zheng, ACS Sens. **2024**, 9 (11), 5782-5791. [17] Z. Zhao, Q. Huang, C. Yan, Y. Liu, X. Zeng, X. Wei, Y. Hu, Z. Zheng, Nano Energy **2020**, 70, 104528.

[18] Y. Hu, Z. Zheng, Nano Energy **2019**, 56, 16-24.

[19] G. Xu, H. Wang, G. Zhao, J. Fu, K. Yao, S. Jia, R. Shi, X. Huang, P. Wu, J. Li, B. Zhang, C. Yiu, Z. Zhou, C. Chen, X. Li, Z. Peng, Y. Zi, Z. Zheng, X. Yu, Sci. Adv. **2025**, 11 (12), eadt0318.

[20] I. Ali, N. Karim, S. Afroj, EcoMat **2024**, 6 (7), e12471.

[21] Y. Liu, C. Yiu, H. Jia, T. Wong, K. Yao, Y. Huang, J. Zhou, X. Huang, L. Zhao, D. Li, M. Wu, Z. Gao, J. He, E. Song, X. Yu, EcoMat **2021**, 3 (4), e12123.

[22] X. Liu, N. Zhang, P. Wang, X. An, J. Shu, Y. Zhu, Y. He, T. Yi, *Energy Storage Mater.* **2024**, *72*, 103742.
